# Evaluating excited state atomic polarizabilities of chromophores†
†Electronic supplementary information (ESI) available: Basis set dependence, definition of bond charges, Romberg differentiation, python script to calculate atomic polarizabilities, influence of the cavity radius, atomic polarizabilities of coumarin 153, all tables in atomic units. See DOI: 10.1039/c7cp08549d


**DOI:** 10.1039/c7cp08549d

**Published:** 2018-03-06

**Authors:** Esther Heid, Patricia A. Hunt, Christian Schröder

**Affiliations:** a University of Vienna, Faculty of Chemistry, Department of Computational Biological Chemistry , Währingerstraße 19 , A-1090 Vienna , Austria . Email: christian.schroeder@univie.ac.at ; Tel: +43 1 4277 52711; b Department of Chemistry, Imperial College London , London , SW7 2AZ , UK . Email: p.hunt@imperial.ac.uk

## Abstract

Ground and excited state dipoles and polarizabilities of the chromophores *N*-methyl-6-oxyquinolinium betaine (MQ) and coumarin 153 (C153) in solution have been evaluated using time-dependent density functional theory (TD-DFT). A method for determining the atomic polarizabilities has been developed; the molecular dipole has been decomposed into atomic charge transfer and polarizability terms, and variation in the presence of an electric field has been used to evaluate atomic polarizabilities. On excitation, MQ undergoes very site-specific changes in polarizability while C153 shows significantly less variation. We also conclude that MQ cannot be adequately described by standard atomic polarizabilities based on atomic number and hybridization state. Changes in the molecular polarizability of MQ (on excitation) are not representative of the local site-specific changes in atomic polarizability, thus the overall molecular polarizability ratio  does not provide a good approximation for local atom-specific polarizability changes on excitation. Accurate excited state force fields are needed for computer simulation of solvation dynamics. The chromophores considered in this study are often used as molecular probes. The methods and data reported here can be used for the construction of polarizable ground and excited state force fields. Atomic and molecular polarizabilities (ground and excited states) have been evaluated over a range of functionals and basis sets. Different mechanisms for including solvation effects have been examined; using a polarizable continuum model, explicit solvation and *via* sampling of clusters extracted from a MD simulation. A range of different solvents have also been considered.

## Abstract 2

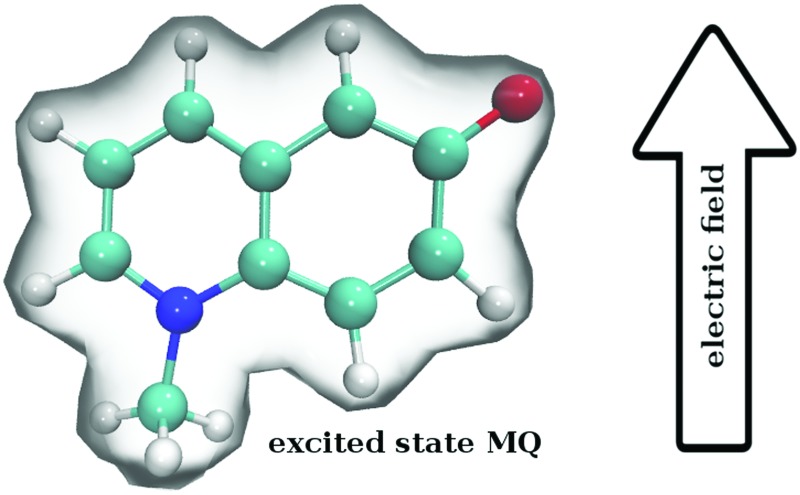
Ground and excited state atomic polarizabilities of the chromophores *N*-methyl-6-oxyquinolinium betaine and coumarin 153 have been evaluated *via* quantum mechanics.

## 1 Introduction

Electronic excitation of a dye molecule (in solution) will usually lead to a dipole moment change, however it is less well recognized that the polarizability can also change. Excitation induced changes in dipole moment and polarizability will lead to changes to the electrostatic and dispersion interactions between solute and solvent.^1^ The surrounding solvent is perturbed and is no-longer in equilibrium. The solvent molecules reorientate and relax reaching a new equilibrium state around the electronically excited solute. Polarizability (as well as dipole) effects can be important, particularly in low-polarity solvents, where dispersion interactions are significant or when the solute is highly polarizable. Moreover, excited states are often more polarizable than the ground state, leading to a considerable difference in the ground and excited state polarizabilities. We are interested in a computational description of the changes in polarizability that occur between the ground and excited state of a solute (dye).

Time-dependent fluorescence spectroscopy measures the timescale of the solute relaxation, indirectly probing the dynamic properties of the solvent.^2^–^10^ The reorganization of the solvent also impacts on the electronic structure of the solute; recently the total (interconnected solute and solvent) response has been directly monitored *via* optical-Kerr-effect spectroscopy.^11^–^13^ The computational description of excitation and relaxation processes often lacks a correct treatment of the solute polarizability and therefore only partly reflects the events probed *via* experiment.

In a computational approach, one would ideally carry out a large scale *ab initio* molecular dynamics (MD) simulation with a post Hartree Fock (HF) method. This level of computation is not yet accessible for realistic dyes and a range of methodologies with different levels of approximation are available. Quantum chemical methods naturally include polarization, coumarin 153 has been studied using an AM1(*i.e.* semiempirical)/MM approach,^14^ and coumarin 120 has been examined at the HF/MM level.^15^ More recently, methodologies sampling solute clusters from classical MD trajectories followed by the evaluation of each cluster using a TD-DFT/MM/PCM approach have been reported.^16^ The next step has been implemented, where a quantum solute region (CAM-B3LYP) is able to polarize the surrounding classical cluster, modeled by a fully polarizable potential (AMOEBA).^17^ Other variations include running *ab initio* MD simulations of various flavors.^18^–^20^

One of the simplest approaches for studying the dynamic solvent response after electronic excitation is to employ classical MD simulations with a fixed partial charge potential for both the solute and solvent. Excitation of the solute is modeled by modifying the partial charges of the solute to represent the excited state electronic distribution.^9^,^10^,^21^–^30^ Note that in this case there is no representation of the impact of the changing electronic structure on the force constants of the solute potential (for bonds, angles or torsion angles) or on the van der Waals terms of the solute potential. Moreover, while the dipole moment may be correctly recovered, the change in the polarizability of the solute is ignored.

The natural next step is to include polarization effects, employing polarizable solute and solvent potentials. Polarizable potentials are available for common solvents but are rare for solutes. Efforts have been made to mimic solute polarizability changes at the classical level, for example using artificial solutes (a rigid diatomic) in MD simulations.^31^–^34^ A number of model (analytic) theories have been developed employing parameters extracted from experimental spectra.^11^,^35^,^36^ Nevertheless, the effect of a change in solute polarizability on the solvent dynamics has not been well studied.

Providing a simple but robust method for determining the ground and excited state polarizability of key chromophores and including polarizability changes within a MD treatment of solvation dynamics is highly desirable. Including solute polarizability is also advantageous from the perspective of providing more accurate trajectories for the mixed quantum mechanics (QM)/molecular mechanics (MM) approaches that sample solute clusters.

Experimentally, the Frank–Condon polarizability change in the solute can be characterized using Stark spectroscopy.^37^–^42^ Relaxed excited state polarizability changes can be probed using time-resolved microwave conductivity.^43^,^44^

Computationally, QM methods provide access to both the ground and excited state electronic structure of a chromophore and can be used to evaluate the polarizability. A more accurate computational model requires the use of either an explicit or implicit solvation model. To access the excited state of larger molecules, TD-DFT can be employed. Recently TD-DFT has been used with a linear response (LR) and a corrected linear response (cLR) Polarizable Continuum Model (PCM) formalism.^45^,^46^ Although the cLR-PCM approach treats the excited state properties more accurately, it has been shown that for many systems the computationally less expensive LR-PCM approach suffices.^45^ Thus, the general procedure outlined in ref. 45 can be used to determine the excited state molecular dipole moments and polarizabilities of solute dyes.

The QM methods described above yield molecular polarizabilities, however, a MD treatment of polarizability usually requires atomic polarizabilities. In MD simulations Drude oscillators can be employed to recover polarizability effects. Charged dummy particles (Drude particles) are attached to each non-hydrogen atom *via* a fictitious harmonic “bond” to mimic the movement of the electron cloud.^47^ The harmonic force constant *kδi* is directly connected to the partial charge of the Drude particle *qδi* and the polarizability *α*_*i*_ of the atom *i via*1
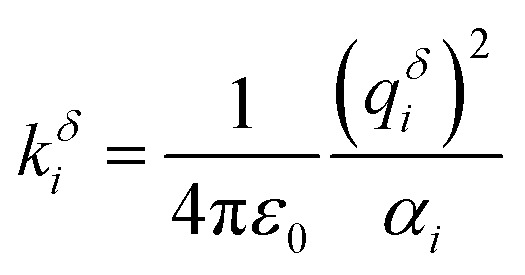
where *kδi* is usually set to a standard value of 500 kcal (mol Å^2^)^–1^. Thus, the polarizability determines the charge of the Drude particle which, in turn, determines the magnitude of the atomic dipole.

To date atomic polarizabilities employed in MD force fields have been calculated *via* a designed regression methodology based on experimental data such as the refractive index and molar volume of similar, pure compounds, usually solvents.^48^–^52^ However, we are not interested in bulk properties of a solvent, but in the electronic properties of a chromophore in a dilute solution. Moreover, refractive indices and molar volumes are not available for electronically excited states, thus only ground state atomic polarizabilities are accessible. A new methodology for decomposing ground and excited state (solution) molecular polarizabilities into atomic contributions based on a computational approach needs to be developed, *i.e.* the decomposition cannot be based on experimental observables. In this study we demonstrate a novel and effective computationally based methodology (employing a set of widely available codes) for determining the atomic polarizabilities of solvated molecules.

**Fig. 1:**
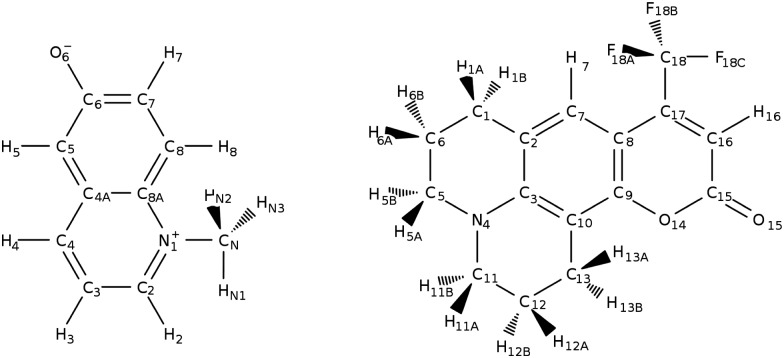
Chromophores and their respective atom labeling used in this study. Left: *N*-Methyl-6-oxyquinolinium betaine, right: coumarin 153.

We consider two well-known molecular probes: *N*-methyl-6-oxyquinolinium betaine (MQ) and coumarin 153 (C153), Fig. 1. For MQ, calculations of the dipole moment in gas phase^9^,^53^,^54^ and solution^9^,^19^ exist, as well as molecular polarizabilities in gas phase.^53^ For C153, atom-type (*i.e.* based on the element, such as C and hybridisation state) polarizabilities have also been computed in the gas phase.^13^ Thus the calculations carried out here will complement the known electronic properties of MQ and C153. More importantly, we will lay the necessary foundations for the general design of polarizable solute force fields.

In this article we outline and test a QM based methodology for determining atomic excited state polarizabilities for (realistic, *i.e.* large) dye molecules in solution. The correct choice of functional and basis set, as well as the influence of the solvent on the calculated electronic properties of the solute are investigated.

## 2 Computational details

All calculations were performed with the Gaussian09 program using density functional theory (DFT).^55^ For all calculations, the energy convergence threshold was improved to 10^–8^ a.u., and the density convergence threshold to 10^–10^ a.u., scf = conver = 10 keyword. An enhanced integration grid was employed (99 radial shells, with 590 angular points per shell), int = ultrafine keyword. Geometry optimization of the ground state was performed with M06-2X, ωB97xD and B3LYP-D3BJ functionals employing a 6-31G(d) basis set, the resulting structures were confirmed as minima by frequency analysis (*i.e.* no imaginary frequencies). TD-DFT was used with the same functionals/basis sets to compute the first excited (singlet) states.

We follow a commonly employed practice of optimizing structures with a modest basis-set and subsequently carrying out single point calculations with a more extensive basis-set. Electronic properties for MQ have been evaluated on the (respective) lower 6-31G(d) level geometry using M06-2X with Sadlej's polarizable pVTZ basis set,^56^ ωB97xD/aug-cc-pVTZ and B3LYP-D3BJ/6-311G(d,p).

The success of TD-DFT methods can depend on the choice of functional, especially for the calculation of excited states with multi-reference character, or those that involve Rydberg states or when strong charge-transfer occurs, in such cases the amount of exchange included can be highly influential.^46^,^57^–^59^ The excitations examined here are simple π to π* transitions without substantial multireference character and only modest charge transfer, thus are not expected to show strong exchange dependent effects.^54^,^60^ To confirm a weak dependence on exchange, the range of functionals outlined above include different levels of exchange; M06-2X (54%), ωB97xD (range sensitive 22–100%) and B3LYP-D3BJ (20%).

Dispersion is important for delocalized π systems, such as the dye molecules examined here. The functionals employed also include a range of mechanisms for modeling dispersion; in the parametrization of the functional (M06),^61^ as a long range corrected hybrid (ωB97xD)^62^ or as an *ad hoc* correction of the B3LYP functional^63^,^64^ *via* the method of Grimme (D3BJ).^65^

Calculations were carried out in the gas phase and with an implicit solvent. A PCM model was employed for water, methanol (MeOH) and ethanol (EtOH).^66^ The ionic liquid, 1-ethyl-3-methylimidazolium-tetrafluoroborate (C_2_mimBF_4_), was modeled *via* the Universal Solvation Model Based on Solute Electron Density (SMD) method.^67^,^68^ In each case, the structure was optimized separately in the gas-phase and solvent environment.

To test the influence of the modest 6-31G(d) basis set on the geometry, we further optimized the structure of MQ employing the M06-2X functional and Sadlej's basis set, both in the gas phase and in PCM (water). No significant differences were found, the root-mean-square deviation of the respective atom coordinates lies under 0.005 Å; further information, including a comparison of the structures can be found in the ESI,† Section 1 and Table S1.

To assess the validity of the implicit solvation model additional calculations were carried out with explicit water molecules. MQ is known to form approximately three hydrogen bonds in the ground state and two hydrogen bonds in the excited state.^60^ Calculations have been carried out with three explicit water molecules positioned near to the oxygen atom within MQ, two different approaches have been applied. In the first method, a conformation in which MQ formed a cluster with three hydrogen bonds was arbitrarily selected from a previously reported MD simulation of MQ in water.^9^ The MQ·3H_2_O cluster was cut and embedded in an implicit (PCM) water solvent and optimized, subsequently the polarizability and dipole moment were computed. In the second method, ten different cluster conformers were extracted from the same MD simulation. MQ and the three water molecules closest to the oxygen atom were cut and the electric structure (polarizability and dipole moment) computed for each frozen conformer (*i.e.* no geometry optimization was carried out at the QM level).

## 3 Results and discussion

### 3.1 Theoretical description

Polarizabilities of the solute ground 
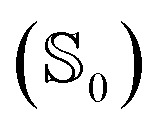
 and excited 
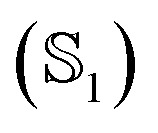
 state can be obtained experimentally or computationally *via* Stark's relation eqn (2) which links the energy *E* of a state to an applied external electric field **F**2
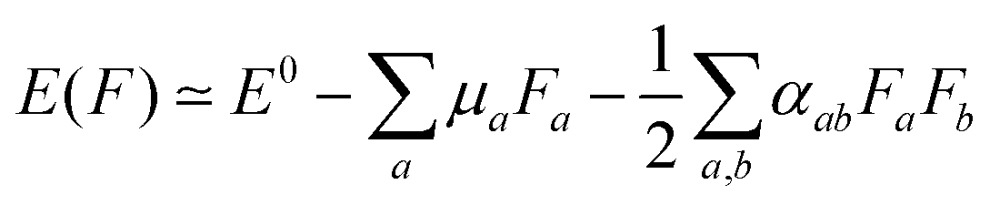
Either the second derivative of the energy or the first derivative of the dipole moment with respect to the electric field can be used to calculate components of the polarizability tensor *α*,3
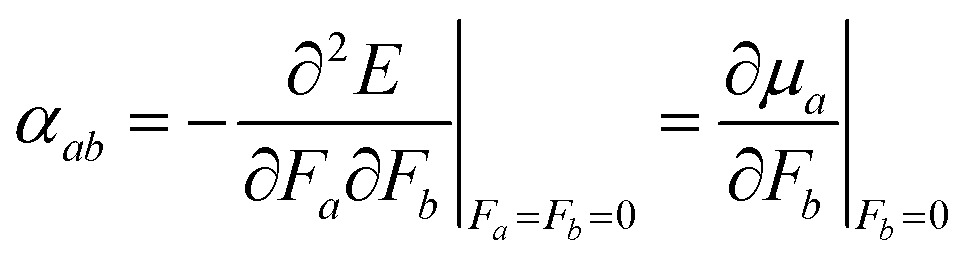
where the subscripts *a* and *b* denote the *x*, *y* and *z* direction. The average polarizability is then defined as the trace of the polarizability tensor *α*,4
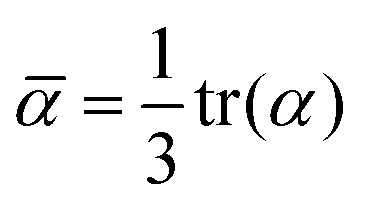
and the polarizability anisotropy *γ* as5
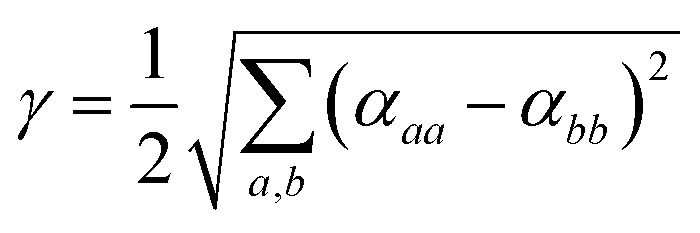



This approach is common for calculating molecular polarizabilities and is valid for both gas phase and implicit solvent calculations of ground or excited state molecules.^1^,^46^,^69^–^72^ However, atomic polarizabilities are required as input for a polarizable MD potential. The atomic polarizability can be calculated as a derivative of the atomic dipole moment with respect to the electric field analogously to eqn (3) and thus we need to determine the atomic dipole moments.

The total molecular dipole moment (for a neutral molecule) can be evaluated relative to an arbitrary reference point, in this case the coordinate system origin is at the center of mass, eqn (6) where *Z*_*i*_ is the nuclear charge, *R*_*i*_ is the nuclear position, *ρ*(*r*) is the electron density and *r* the electronic coordinate:6
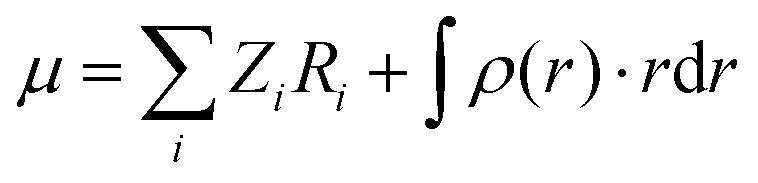



The total dipole moment *μ*, eqn (6), can also be thought of in terms of a sum of atomic dipole contributions, eqn (7). Atomic charges and atomic dipoles can be defined on each atom (within a non-overlapping atomic integration basin *Ω*_*i*_), eqn (8).7
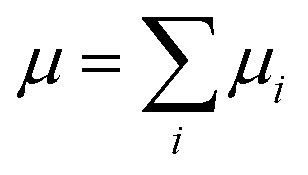

8
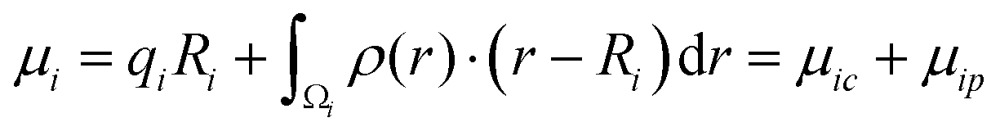



The atomic charge *q*_*i*_ includes the nuclear charge and some localized surrounding electron density. Within an atomic basin electron density can polarize and contribute to an atomic dipole moment *μ*_*ip*_. Thus, *q*_*i*_ is not the “normal” atomic charge which is associated with a zero atomic dipole moment. We will now call *μ*_*ic*_ the atomic dipole charge contribution and *μ*_*ip*_ the atomic dipole polarization contribution.

When an electric field is applied to a molecule the distribution of charge is perturbed, electron density can be transferred from one atom to another and the atomic positions *R*_*i*_ can change giving rise to Δ*μ*_*ic*_. The electron density distribution around an atomic site will change and the atomic basin defining the volume of the atom can also change, (resulting in Δ*Ω*_*i*_) giving rise to Δ*μ*_*ip*_.^73^

For each atomic contribution the charge term depends on the absolute position of the atom (while the polarization term does not, since it describes only the relative positions of nucleus and electron cloud). To avoid this dependence, partial charges (on atomic sites *i* in a neutral molecule) can be converted to a sum of surrounding bond charges *q*_*b*(*ij*)_ where *j* is an adjacent bonded atomic site and the coordinate contribution to *μ* is defined relative to the atomic center.^74^,^75^
9
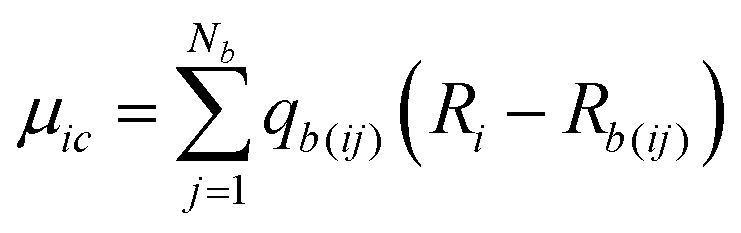



Thus, *q*_*b*(*ij*)_ is the contribution to the net charge of *i* from the directed bond between the atoms *i* and *j*,^74^*N*_*b*_ is the number of bonds or bond critical points connected to atom *i* and *R*_*b*(*ij*)_ is the charge position. This can be at a bond critical point between atom *i* and *j*, however *R*_*b*(*ij*)_ can also simply be mid-way between the atoms, 
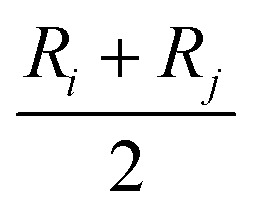
. The net charge on bond points *q*_*b*(*ij*)_ must add up to the atomic dipole charge contribution of the respective atom, and *q*_*b*(*ij*)_ also makes a contribution to atom *j*.10
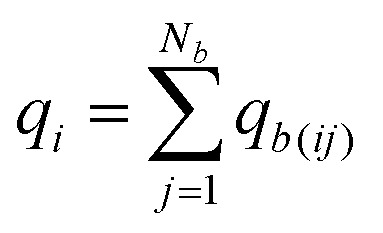



Since *q*_*b*(*ij*)_ describes directed contributions, a reversal of *i* and *j* leads to a change in sign, *q*_*b*(*ij*)_ = –*q*_*b*(*ji*)_. If a ring is present in the molecule, the sum of all bond charges in the ring is zero. To determine the bond point charges a set of linear equations is set up (and solved) based on the chemical bonding and the physical restrictions defined. More details and an example are provided in the ESI,† Section 2.

Applying an electric field and employing finite difference methods, the atomic dipole charge and atomic dipole polarizability changes can be evaluated.11
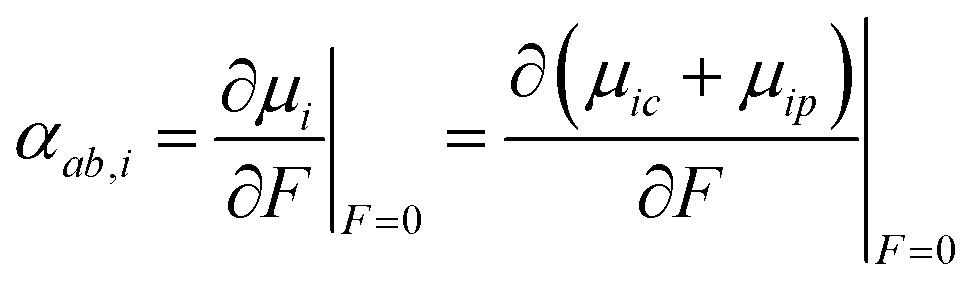
We employ a Romberg differentiation procedure, eqn (12), which uses a geometric sequence 2^*k*^*h* with *k* = 1, 2… and a recursive formula to determine the differential, where *p* is the number of the Romberg iteration and *P* the current differential.^76^12
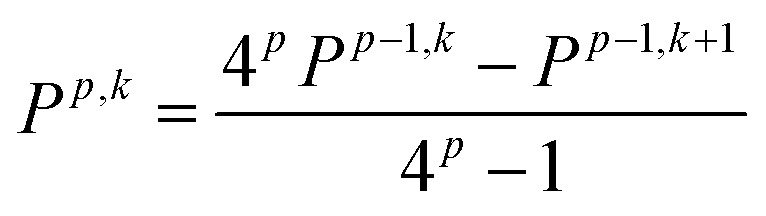
To determine the accuracy at different values of *k*, the Romberg procedure was tested on a single molecule, details are provided in the ESI,† Section 3. Successively more accurate evaluations of *α* up to *k* = 5 were determined. Testing ascertained that an accurate value of the polarizability (within Δ*α* ± 0.01 Å^3^) can be obtained for *k* = 1.

For example, for a specific atom the atomic dipole polarization (*μ*_*ip*_) is evaluated under a positive (and negative) field in the *x*-direction delivering *μ*_*x*,*ip*_(*F*_*x*_) and *μ*_*x*,*ip*_(–*F*_*x*_) respectively. Then simple finite difference yields the polarization tensor component *α*_*xx*,*ip*_ for that atom, eqn (13).13
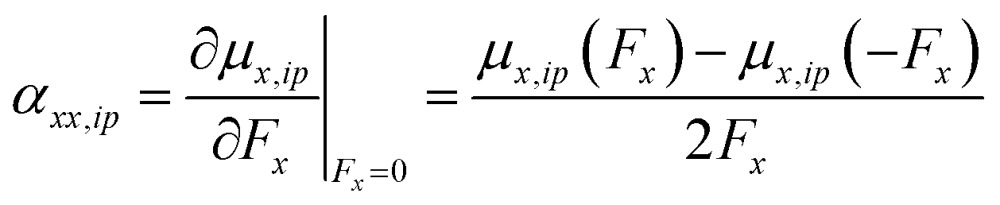



A similar procedure is applied to all the diagonal tensor components and used to determine the average polarizability for that atom 
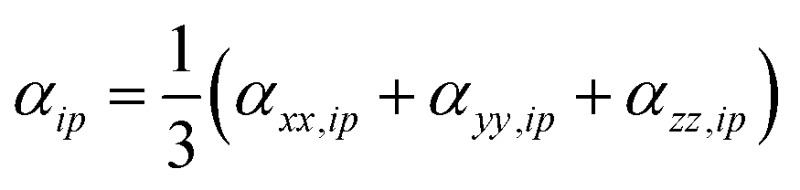
. Subsequently the molecular polarizability can be calculated as the sum of the contributions from each atom,14
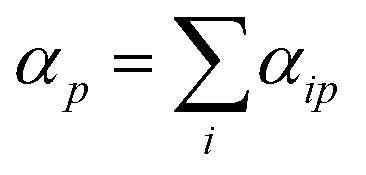



Once the atomic and molecular polarizabilities for the 
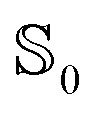
 and 
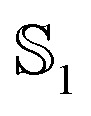
 state are known, they can be used to develop a polarizable ground and excited state force field for chromophores.

### 3.2 Implementation

Most electronic structure codes will deliver the molecular dipole moment and the molecular polarizability tensor *via* an analytic or numeric calculation (in G09 use the “polar” keyword). Atomic dipole charges (*q*_*i*_) and the atomic dipole polarization (*μ*_*ip*_) were obtained from the GDMA code of Misquitta and Stone (multipole moments of rank 1).^77^,^78^

These quantities are then re-evaluated under the influence of an electric field. A simple vector summation of the atomic bond charges, evaluated with the field on and off, forms the charge transfer component (*α*_*ic*_) of the atomic polarizability. The average polarization component (*α*_*ip*_) is obtained by finite difference methods; only the diagonal components are needed for each atom. Evaluating *α*_*i*_ for *k* = 1 therefore requires only 6 additional calculations; we applied an electric field of magnitude 0.0008 a.u. in each of the positive and negative *x*, *y* and *z* directions. The molecular polarizability is then obtained by summing the atomic dipole charge transfer and atomic dipole polarizability components. A python script (ESI,† Section 4) was developed to calculate the atomic polarizabilities (*i.e.* the atomic dipole charge transfer and atomic dipole polarizability) from the atomic coordinates, charges and dipoles.

### 3.3 Choice of method: functional and basis set

Dipole moments and polarizabilities for MQ in the gas phase and implicit water for different functionals and basis sets are reported in Table 1. TD-DFT has previously been shown to yield accurate electronic properties for MQ, where it was tested against more elaborate methods such as CIS, ROKS and EOM-CCSD.^54^ The 
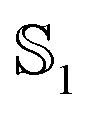
 state computed using BLYP was dark and thus *T*_1_ was computed in ref. 54. Recently, TD-DFT employing a PCM solvent was successfully used to calculate absorption and emission spectral shapes of MQ, where it was also confirmed *via* CASSCF/CASPT2 that the first excited state is a single HOMO–LUMO excitation with no multireference character.^60^ Thus, we expect TD-DFT (in general) to yield an accurate description of both the ground and excited state electronic properties of MQ.

**Table 1:** Dipole moments *μ* in [D] and polarizabilities *α*, as well as polarizability anisotropy *γ* in [Å^3^] of MQ in gas phase and water using different functionals, basis sets and literature values. Dipole moments and polarizabilities in [a.u.] are given in the ESI, Section 7 and Table S4

	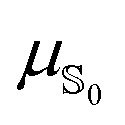	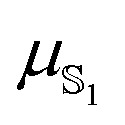	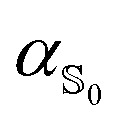	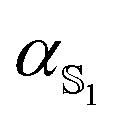	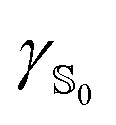	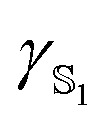
*In vacuo*:						
M06-2X/Sadlej	10.8	6.9	22.0	20.5	17.6	13.4
ωB97xD/aug-cc-pVTZ	11.0	6.8	22.0	20.3	17.7	13.5
B3LYP-D3BJ/6-311G(d,p)	10.1	7.2	19.8	17.5	19.0	14.2
CASSCF/cc-pVTZ^53^	10.5	5.8	21.2	19.1		
BLYP-LSD T_1_/6-311G**^54^	10.2	6.8				
ωB97xD/aug-cc-pVTZ^9^	11.1	7.1				

PCM water:						
M06-2X/Sadlej	16.9	9.7	30.9	31.7	25.2	24.5
ωB97xD/aug-cc-pVTZ	17.1	9.7	30.9	32.0	25.0	25.3
B3LYP-D3BJ/6-311G(d,p)	15.3	9.3	27.7	24.5	28.2	21.1
BLYP/pVTZ^19^	22	14				
ωB97xD/aug-cc-pVTZ^9^	16.5	8.6				

The calculated dipole moments and polarizabilities in gas phase correspond well to those reported in literature. In solution slight deviations from the literature values occur. The dipole moments in ref. 19 were calculated from an *ab initio* trajectory of MQ and explicit water molecules, deviations might therefore stem from the influence of an explicit solvent. In ref. 9, geometry optimization was only carried out in gas phase in the ground state, thus deviations might stem from changes in geometry upon solvation or excitation.

The B3LYP-D3BJ/6-311G(d,p) method produces a low dipole moment for the vacuum ground state, as well as for both solvated states and also yields low polarizabilities, especially for the excited state, when compared to the other methods examined in Table 1. Thus, B3LYP-D3BJ/6-311G(d,p) may be failing to describe the charge-transfer correctly. The same picture arises for the anisotropy of the polarizability. For both the ground and excited state, *γ* obtained from M06-2X/Sadlej corresponds well to that obtained from ωB97xD/aug-cc-pVTZ, whereas B3LYP-D3BJ/6-311G(d,p) shows much larger deviations. It appears therefore that B3LYP-D3BJ/6-311G(d,p) may not be a suitable method for the calculation of molecular or atomic polarizabilities. We find that M06-2X/Sadlej provides comparable results to the more elaborate ωB97xD/aug-cc-pVTZ level of theory, hence we employ the less expensive M06-2X/Sadlej in all further calculations.

### 3.4 Influence of the solvent model

Dipole moments and polarizabilities for MQ and C153 in the 
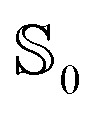
 and 
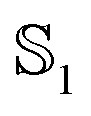
 state evaluated (using M06-2X/Sadlej) with different solvents and different solvent models are reported in Table 2. The vacuum polarizability of C153 in the ground and excited state corresponds well to the literature values of 28.2 Å^3^ and 34.4 Å^3^, respectively.^13^ Polarizabilities of MQ were already compared to literature values in the previous subsection.

**Table 2:** Dipole moments in [D] and polarizabilities in [Å^3^] for MQ and C153 in different solvents using the PCM and SMD implicit solvent models. For the explicit water models (termed ‘expl.’), ‘opt.’ refers to a calculation at a single, optimized geometry, whereas ‘rep.’ refers to the average over ten snapshots from MD simulations without optimization (95% confidence interval given). Dipole moments and polarizabilities in [a.u.] are given in the ESI, Section 7 and Table S5

	*ε*	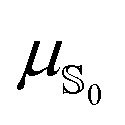	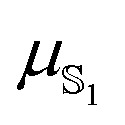	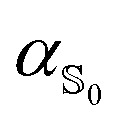	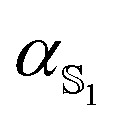	Δ*α* (%)
MQ:						
Vacuum	1	10.8	6.9	21.9	20.4	–7
PCM water	78.4	16.9	9.7	30.8	31.7	+3
PCM MeOH	32.6	16.7	9.6	30.5	31.3	+2
PCM EtOH	24.9	16.6	9.5	30.4	31.0	+2
PCM C_2_mimBF_4_	12.9	16.2	9.3	29.8	30.1	+1
SMD water	78.4	19.2	11.1	23.9	36.3	+10
SMD C_2_mimBF_4_	12.9	17.5	10.1	31.7	33.3	+5
Expl. + PCM opt.	78.4	14.5	8.4	29.8	32.5	+9
Expl. + PCM rep.	78.4	14.2	8.4	31.4	32.5	+9
		±1.3	±1.0	±0.6	±0.7	

C153:						
Vacuum	1	7.0	12.4	31.6	34.8	+10
PCM water	78.4	10.0	18.9	45.5	48.2	+6
PCM MeOH	32.6	9.9	18.7	44.9	47.6	+6
PCM EtOH	24.9	9.9	18.5	44.6	47.3	+6
SMD C_2_mimBF_4_	12.9	10.2	19.4	46.7	49.0	+5

The data (in solution) reported in Table 2 all correspond to default PCM radii (scaled VdW-radii, scaling factor 1.1 for PCM and scaling factor 1.0 for SMD) The influence of the PCM radii was tested and it was found that an increased radius slightly influences dipole moment and polarizability. A larger radius will push the surface charges at the solvent cavity boundary further from the atomic centers, weakening Coulombic interactions and thus will act similarly to a reduction in the dielectric constant of the solvent. Further information and data is provided in the ESI,† Section 5 and Table S2.

In general, the lower the dielectric constant of the solvent, the smaller the absolute dipole moment and polarizability, for both the ground and excited states. Nevertheless, the effect is very small and changes are similar for both states. There is a slight difference between water modeled using PCM *vs.* SMD probably due to the different parametrization employed for each method. For all the solvents studied using PCM, the ratio between 
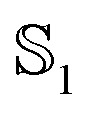
 and 
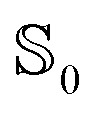
 state polarizability remains nearly unchanged at a value of about 1.02 for MQ and 1.06 for C153. Importantly, the similar ratios over a range of different solvents indicate that a single force field (for each chromophore) could be applicable in a wide range of solvents.

The vacuum dipole moment and polarizability of MQ are very low and decrease upon excitation, in contrast to the solvent environments where both MQ and C153 exhibit a polarizability increase upon excitation. Thus, these results (for MQ and C153) indicate that gas phase calculations should not be employed to predict polarizability in solution.

**Fig. 2:**
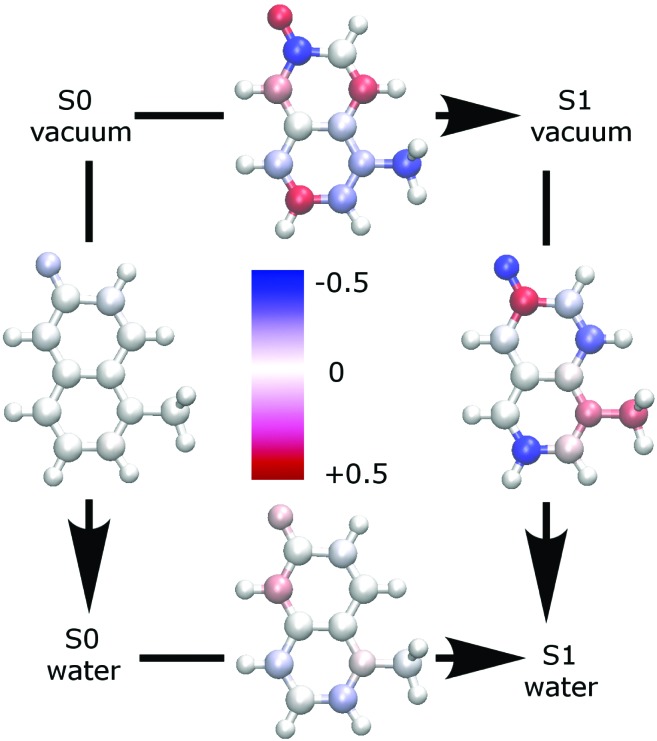
Partial charge change upon change of state or solvation, evaluated using CHelpG at the ωB97xD/aug-cc-pVTZ level. Charge changes more positive than 0.5 (red) or more negative than –0.5 (blue) are shown in the same color as ±0.5.

Fig. 2 shows the change of partial charges upon inclusion of a solvent (water) or on excitation. Ground state properties are minimally influenced by an implicit solvent (left panel of Fig. 2), while the excited state properties are more substantially impacted (right panel of Fig. 2).^79^,^80^ The inclusion of solvent effects is therefore very important to yield the correct solvated excited state electric properties. Furthermore, changes in the partial charges observed upon excitation in the gas phase (top panel of 2) appear to have too high values which are physically implausible.

Table 2 also lists the dipole moment and polarizability for a cluster of one MQ molecule and three water molecules located close to the oxygen atom evaluated for an optimized geometry (opt.) or from frozen geometries taken as snap-shots from a MD simulation (rep.). The QM calculation reports a whole cluster dipole moment and polarizability, thus to determine the dipole moment and polarizability of the individual molecules we use the CHelpG charges. Dipole moments are calculated classically *via*
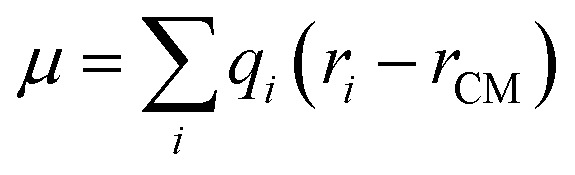
 where *q*_*i*_ are the respective partial charges and *r*_CM_ is the position of the center of mass of MQ (without water). The total charge of MQ is not zero, as some of the electron density has moved into the H-bonded water molecules. Thus, because the charge is slightly reduced the dipole moments obtained are estimates (relative to the other values reported). Polarizabilities have been calculated by subtracting three times the polarizability of a single water molecule (in PCM water) from the overall values, and thus these values are also only estimates, (relative to the other values reported).

The ground and excited state polarizabilities of MQ are similar for the optimized MQ·3H_2_O cluster, and for the ten averaged MD conformations (within the evaluated uncertainty in *α*), compared to MQ within an implicit water solvent. Furthermore, the geometry of MQ differs between the ten MD snapshots and the optimized cluster, while the polarizabilities do not. These results support the use of fixed polarizabilities within a MD potential, and indicate that explicit solvent molecules do not strongly influence the overall polarizability in this system. However, an analysis of the precise influence of explicit solvent on the polarizability would require a larger number of snapshots. Alternatively, the cluster polarizability can be dissected into atomic contributions, and summed up over all solute contributions to yield the solute polarizability. The calculation of atomic contributions furthermore enables the detection of site-specific changes in polarizability induced by hydrogen bonds that are lost in the overall molecular property.

### 3.5 Atomic polarizability

We have established that the M06-2X functional with a Sadlej basis set and PCM (or explicit) solvation model is appropriate and we now proceed to use this methodology for calculating the atomic polarizabilities of MQ, MQ·3H_2_O and C153. The atomic polarizability was calculated as the change in atomic dipole moment upon applying an external field (here 0.0008 a.u.), where there is an atomic dipole contribution from charge transfer *μ*_*c*_ arising from a change in partial charge at each atomic site and an atomic dipole polarization contribution *μ*_*p*_, arising from the change in inherent dipole moment at each atomic site.

The respective atomic polarizabilities for MQ and MQ·3H_2_O are given in Table 3 (atom labeling is taken from Fig. 1), the corresponding values for C153 are given in the ESI,† Section 6 and Table S3.

**Table 3:** Atomic polarizability *α*_*i*_ and the contributions from polarization, *α*_*p*,*i*_ and charge transfer, *α*_*c*,*i*_ of MQ and MQ·3H_2_O in PCM water in the ground and excited state. Δ*α*_*i*_ describes the change of atomic polarizability upon excitation. Atom labeling as in Fig. 1. Atomic polarizabilities in [a.u.] are given in the ESI, Section 7 and Table S5

	MQ in PCM water							MQ·3H_2_O in PCM water						
	Ground state			Excited state			Δ*α*	Ground state			Excited state			Δ*α*
*i*	*α*_*p*,*i*_	*α*_*c*,*i*_	*α*_*i*_	*α*_*p*,*i*_	*α*_*c*,*i*_	*α*_*i*_	Δ*α*_*i*_ (%)	*α*_*p*,*i*_	*α*_*c*,*i*_	*α*_*i*_	*α*_*p*,*i*_	*α*_*c*,*i*_	*α*_*i*_	Δ*α*_*i*_ (%)
C_N_	0.33	0.86	1.19	0.33	0.93	1.26	+6	0.33	0.86	1.19	0.33	0.93	1.26	+7
H_N1_	0.24	0.15	0.40	0.27	0.16	0.43	+9	0.24	0.15	0.39	0.27	0.16	0.43	+10
H_N2_	0.24	0.13	0.37	0.28	0.15	0.43	+17	0.22	0.13	0.36	0.28	0.15	0.41	+16
H_N3_	0.24	0.13	0.37	0.28	0.15	0.43	+17	0.22	0.13	0.36	0.28	0.15	0.41	+17
N_1_	0.30	2.00	2.30	0.39	2.02	2.40	+5	0.30	1.87	2.16	0.39	2.04	2.43	+12
C_2_	0.59	1.36	1.96	0.74	1.42	2.16	+11	0.53	1.29	1.82	0.71	1.44	2.15	+18
H_2_	0.27	0.22	0.49	0.33	0.24	0.56	+15	0.25	0.21	0.46	0.33	0.24	0.55	+21
C_3_	0.64	1.33	1.96	0.73	1.45	2.18	+11	0.64	1.29	1.93	1.45	0.71	2.16	+12
H_3_	0.30	0.22	0.50	0.33	0.22	0.55	+8	0.28	0.21	0.49	0.31	0.22	0.55	+10
C_4_	0.55	1.69	2.24	0.70	1.76	2.46	+10	0.52	1.59	2.09	0.70	1.78	2.47	+18
H_4_	0.27	0.18	0.44	0.33	0.21	0.52	+17	0.25	0.18	0.43	0.31	0.19	0.50	+18
C_4A_	0.39	2.30	2.70	0.49	2.49	2.98	+10	0.40	2.12	2.52	0.47	2.52	2.99	+19
C_5_	0.73	1.94	2.68	0.55	1.90	2.45	–8	0.67	1.76	2.43	0.53	1.90	2.43	+0
H_5_	0.31	0.19	0.50	0.27	0.18	0.44	–13	0.21	0.15	0.36	0.16	0.13	0.31	–15
C_6_	0.36	2.12	2.46	0.34	1.93	2.27	–8	0.33	2.04	2.37	0.31	2.06	2.36	+0
**O**_6_	**1.48**	**0.73**	**2.21**	**1.20**	**0.68**	**1.88**	**–15**	**0.87**	**1.41**	**2.28**	**0.79**	**1.22**	**2.00**	**–12**
C_7_	0.65	1.54	2.19	0.76	1.57	2.33	+7	0.59	1.39	1.99	0.76	1.60	2.19	+18
H_7_	0.33	0.22	0.55	0.34	0.24	0.58	+5	0.19	0.16	0.36	0.30	0.22	0.52	+46
C_8_	0.59	1.73	2.31	0.58	1.81	2.39	+3	0.58	1.60	2.18	0.58	1.84	2.42	+11
H_8_	0.24	0.16	0.41	0.24	0.16	0.40	–2	0.24	0.16	0.40	0.24	0.16	0.40	+0
C_8A_	0.43	2.19	2.61	0.39	2.30	2.70	+3	0.40	2.03	2.43	0.39	2.33	2.71	+12

Total	9.3	21.5	30.8	9.8	21.9	31.7	+3	8.4	20.7	29.0	9.2	22.7	31.9	+10

We first consider MQ in PCM water. The charge transfer *α*_*c*_ for the whole molecule is 21.5 Å^3^ in the 
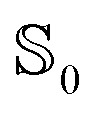
 state and 21.9 Å^3^ in the 
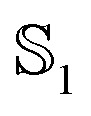
 state. The dipole polarizability *α*_*p*_ is 9.3 Å^3^ in the 
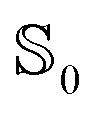
 state and 9.8 Å^3^ in the 
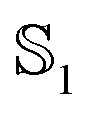
 state. Both contribute to the molecular polarizabilities reported in Table 2. Thus, the charge transfer term accounts for most of the molecular polarizability. Dominance by the charge transfer term has been determined previously for other compounds, *e.g.* nitroanilines.^81^

**Fig. 3:**
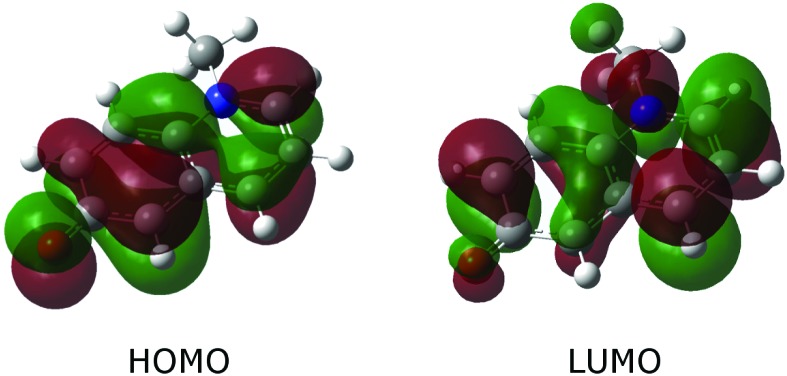
Highest occupied and lowest unoccupied orbital of MQ. The ES is a pure HOMO–LUMO transition.

A closer look at Table 3 and at Fig. 3, which shows the highest occupied (HOMO) and the lowest unoccupied (LUMO) molecular orbital, reveals that the change in polarizability is related to the change of electron density distribution upon excitation. The bottom panel of Fig. 2 also shows the charge redistribution.

Electron density from the phenyl ring, mostly from C_5_, O_6_ and C_6_ is shifted towards the pyridinium part of MQ; C_5_, O_6_ and C_6_ all show a decreased polarizability with respect to both polarization and charge transfer components. The sites gaining electron density, mostly C_2_ and C_4_, show an increased polarizability. This finding corresponds with our natural expectation that the electron cloud at an electron rich site is more likely to move upon perturbation than the tightly bound electron cloud around an electron deficient site. These changes in polarizability affect the intrinsic polarization *α*_*ip*_ to a larger extent but have also a minor contribution from the charge transfer term *α*_*ic*_. Overall, *α*_*ip*_ changes more upon excitation than *α*_*ic*_ (up to ±29% compared to ±12%), but in absolute terms *α*_*ic*_ contributes about two thirds to the total atomic polarizability, so that the overall change in atomic polarizability at a site does not exceed ±17% in MQ. The computed change in the molecular polarizability, +2%, does not reflect the local site-specific change in atomic polarizability, ±17%.

The insight obtained from studying the individual atomic polarizabilities is important; the atomic polarizability does not uniformly increase upon excitation. This means an excited state potential cannot be developed by simply scaling the molecular ground state polarizabilities with 
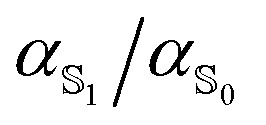
. Polarizability changes are very site and structure specific. Transferability of atomic polarizabilities should also not be taken for granted, especially for specific functional groups within a molecule.

For example, polarizabilities from various designed regression calculations fail to reproduce atomic polarizabilities of strongly charged sites. In MQ, the anionic oxygen O_6_ (highlighted in bold in Table 3) shows a much larger polarizability (1.48 Å^3^ from polarization, 2.21 Å^3^ in total) with respect to literature atomic polarizabilities for this atom type (oxygen), 0.3 to 0.7 Å^3^.^48^–^52^ The failure of atomic polarizabilities from designed regression was already reported for charged molecules,^48^ but not for charged atom sites. The corollary is that experimental evaluations of transferable atomic atom type polarizabilities *via* designed regression should only be carried out for very similar molecular classes.

The effects of explicit solvation can be evaluated by considering changes in the ground state atomic polarizability of O_6_. Three water molecules solvate the O_6_ site and (the ground state) polarization contribution decreases from 1.48 Å^3^, to 0.87 Å^3^, while at the same time the charge transfer polarization increases from 0.73 Å^3^, to 1.41 Å^3^. The presence of the hydrogen bonds has increased the charge on the oxygen atom while also hindering the free movement of the electron density and reducing polarization. Thus these results indicate that for an accurate representation of the atomic polarizabilities of solvated molecules, one should include explicit solvent molecules when hydrogen bonding is moderate to strong.

The overall molecular polarizability decreases in the ground state (with three hydrogen bonds) and increases slightly in the excited state (with two hydrogen bonds), so that the overall change for explicit solvation is larger than for isolated MQ. The contributions from polarization and charge transfer for MQ in MQ·3H_2_O (29.0 Å^3^ in the ground state and 31.9 Å^3^ in the excited state) do not correlate directly with the molecular polarizabilities reported in Table 2 (29.8 Å^3^ and 32.5 Å^3^ respectively). The latter are only estimates, as they were calculated *via α*(MQ·3H_2_O) – 3*α*(H_2_O). Thus, the calculation of atomic contributions also ensures the correct distribution of the overall polarizability of a system to the respective atomic sites and individual molecules within a cluster. An accurate decomposition of the cluster polarizability into molecular components is especially important for larger systems, *e.g.* where a larger number of solvent molecules are included explicitly.

For C153 (ESI,† Section 6 and Table S3), a different picture arises: the atomic polarizabilities for all the aromatic C atoms and the non-aromatic ring C atoms is roughly the same. Only C_18_, the fluorinated carbon atom has a very low polarizability, which shows the influence of the electronegative fluorine atoms. Upon excitation, the polarization increases; now some of the ring C atoms show a larger increase in polarizability than other atoms, nevertheless the difference is not as extreme as in MQ.

The high-frequency dielectric constant *ε*_∞_ of the chromophore in its PCM cavity can be evaluated using the following relation,15
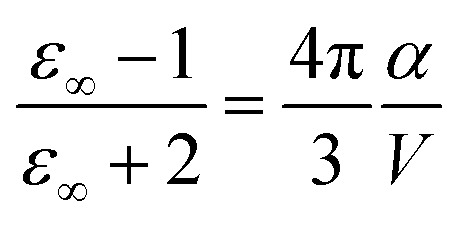
where the polarizability *α* is from Table 3 and the volume of the PCM cavity has been employed. The dielectric constants and PCM cavity volumes for MQ and C153 are given in Table 4. For MQ *ε*_∞_ is 4.9 in the ground and 5.2 in the excited state respectively, these correspond well with the empirical value of 5.3.^82^ Upon excitation, both the *ε*_∞,*p*_ polarization and *ε*_∞,*c*_ charge transfer contributions increase. While contributions to the polarizability are additive, contributions to the dielectric constant are not, thus *ε*_∞_ ≠ *ε*_∞,*c*_ + *ε*_∞,*p*_. For C153 the dielectric constant is 4.2 in the ground and 4.5 in the excited state. Although the polarizability in C153 is larger than in MQ, the increased volume in C153 counteracts this effect, so that the dielectric constant is slightly smaller than in MQ.

**Table 4:** Dielectric constant *ε*_∞_ and the respective contributions from polarization, *ε*_∞,*p*_ and charge transfer, *ε*_∞,*c*_. The volume *V* of the PCM cavity is given in [Å^3^]

	MQ		C153	
	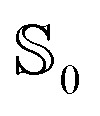	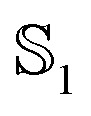	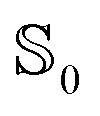	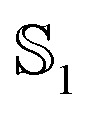
*ε*_∞_	4.9	5.2	4.2	4.5
*ε*_∞,*p*_	1.6	1.7	1.5	1.5
*ε*_∞,*c*_	2.9	3.0	2.8	2.9
*V*	227.8	228.4	371.3	372.0

## 4 Conclusion

The ground and excited state molecular dipole moments and polarizabilities of the fluorescence probes *N*-methyl-6-oxyquinolinium betaine and coumarin 153 in different solvents have been computed. The ratio 
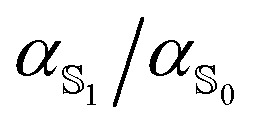
, was found to be approximately 1.02 for MQ, and 1.06 for C153.

Use of the exchange–correlation functional M06-2X with the Sadlej polarizable pVTZ basis set was found to be suitable for the calculation of excited state properties, yielding analogous results to the more elaborate ωB97xD/aug-cc-pVTZ method, but at a much lower computational cost.

The influence of the solvent is crucial; for MQ in the gas phase the polarizability is reduced on excitation, while in the solvent the polarizability is increased (on excitation). Thus, employing a gas-phase calculation to predict solution phase polarizabilities can deliver qualitatively incorrect results. Upon the inclusion of explicit water molecules, the atomic polarizability at the oxygen site of MQ decreases significantly. For an accurate representation of atomic polarizabilities it is therefore necessary to include hydrogen bonding partners where appropriate.

Dissection of the overall molecular polarizability of MQ, MQ·3H_2_O and C153 in PCM water into atomic contributions provided insight into the site specific changes of electric properties upon excitation, as well as the influence of hydrogen bonding on the atomic polarizability. On excitation, the phenyl ring of MQ looses electron density and becomes less polarizable, whereas the pyridinium ring gains electron density and shows increased polarizability at all sites. The polarizability changes of C153 are less site-specific upon excitation. Therefore, the polarizability of some chromophores does not uniformly increase upon excitation and the transferability of atomic polarizabilities should be treated carefully. Furthermore, the change of atomic polarizability upon excitation is not reflected in the molecular ratio 
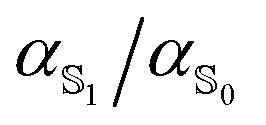
 at many sites.

To sum up, the quantum mechanical calculation of atomic polarizabilities provides a good description of molecular electronic structure in the systems studied and enables the set-up of accurate ground and excited state polarizable force fields, through the calculation of atomic polarizability components.

## Conflicts of interest

There are no conflicts to declare.

## Supplementary Material

Supplementary information
