# Supplementary material for: Evaluating excited state atomic polarizabilities of chromophores
Source: Phys Chem Chem Phys. 2018 Mar 6;20(13):8554–63. doi: 10.1039/c7cp08549d (PMC5885803; doi:10.1039/c7cp08549d)
Supplement: Supplementary file 1 [file CP-020-C7CP08549D-s001.pdf]

Electronic Supplementary Material (ESI) for Physical Chemistry Chemical Physics.
This journal is © the Owner Societies 2018

Supporting information for:

Evaluating Excited State Atomic Polarizabilities of Chromophores

Esther Heid[1], Patricia Hunt[*2], and Christian Schröder[†1]

[1] *University of Vienna, Faculty of Chemistry, Department of Computational Biological Chemistry, Währingerstraße 19, A-1090 Vienna, Austria*
[2] *Department of Chemistry, Imperial College London, London, SW7 2AZ, UK*

[*] p.hunt@imperial.ac.uk
[†] christian.schroeder@univie.ac.at

1 Basis-set dependence of optimized structures

For optimization, only a 6-31G(d) basis set was employed instead of Sadlej's polarizable pVTZ basis set. We tested the resulting differences in structure by calculating optimized geometries using both basis sets. Since deviations are too small to be visible, we calculated the root-mean-square deviation (RMSD), defined as

$$RMSD = \sqrt{\frac{1}{N}\sum_{i=1}^{N} ||v_i - w_i||^2} \quad (1)$$

between two coordinate sets v (6-31G(d)) and w (Sadlej) to describe the induced changes, Table S1. Table S1 furthermore lists the three largest deviations in bond length and angle for all four systems. No significant difference could be found. The corresponding coordinates are given in the following.

Table S1: RMSD of MQ, as well as three largest deviations in bond lengths, Δb, and angles, Δa, between geometry optimizations using a 6-31G(d), or Sadlej's polarizable pVTZ basis set.

	bond	Δb	angle	Δa	RMSD
$\mathbb{S}_0$ vacuum	C_N-H_{N2}	0.005 Å	C_5-C_6-C_7	0.44°	0.0040 Å
	C_N-H_{N3}	0.005 Å	H_{N2}-C_N-H_{N3}	0.35°	
	C_6-C_7	0.005 Å	H_5-C_5-C_6	0.32°	
$\mathbb{S}_0$ water	C_6-O_6	0.011 Å	C_5-C_6-C_7	0.67°	0.0043 Å
	C_5-C_6	0.006 Å	H_5-C_5-C_6	0.49°	
	C_{4A}-C_5	0.006 Å	C_6-C_7-C_8	0.42°	
$\mathbb{S}_1$ vacuum	N_1-C_{8A}	0.006 Å	N_1-C_2-H_2	0.46°	0.0047 Å
	C_{4A}-C_5	0.005 Å	H_2-C_2-C_3	0.43°	
	C_7-H_7	0.004 Å	C_4-C_{4A}-C_{8a}	0.34°	
$\mathbb{S}_1$ water	N_1-C_{8A}	0.008 Å	C_4-C_{4A}-C_{8A}	0.39°	0.0055 Å
	C_{4A}-C_{8A}	0.006 Å	N_1-C_2-H_2	0.34°	
	C_{4A}-C_5	0.006 Å	H_2-C_2-C_3	0.32°	

Optimized geometry of ground state MQ in gasphase, at the M06-2x/6-31G(d) level of theory

```
   21
scf done:   -516.197587
 C     -1.211165      0.000000      2.156737
 C     -0.010394      0.000000      1.387045
 C      1.228356      0.000000      2.135306
 N      1.189150      0.000000      3.500786
 C      0.041763      0.000000      4.197242
 C     -1.184142      0.000000      3.528225
 C      2.456417      0.000000      1.417219
 C      2.443610      0.000000      0.056666
 C      1.217454      0.000000     -0.762918
 C      0.001226      0.000000      0.004696
 O      1.279843      0.000000     -1.996288
 C      2.452541      0.000000      4.248603
 H      2.229069      0.000000      5.313525
 H      3.027382      0.891792      3.994717
 H      3.027382     -0.891792      3.994717
 H      0.127539      0.000000      5.274970
 H     -2.096330      0.000000      4.112819
 H     -2.159524      0.000000      1.627419
 H     -0.938466      0.000000     -0.538857
 H      3.377958      0.000000     -0.497741
 H      3.405341      0.000000      1.942376
```

Optimized geometry of ground state MQ in gasphase, at the M06-2x/Sadlej level of theory

```
   21
scf done:   -516.323589
 C     -1.212351      0.000000      2.155605
 C     -0.010498      0.000000      1.389904
 C      1.226352      0.000000      2.135906
 N      1.189262      0.000000      3.499786
 C      0.044877      0.000000      4.194806
 C     -1.184341      0.000000      3.528429
 C      2.455046      0.000000      1.419892
 C      2.445241      0.000000      0.057124
 C      1.218344      0.000000     -0.757531
 C      0.000534      0.000000      0.003356
 O      1.281646      0.000000     -1.993883
 C      2.450284      0.000000      4.244560
 H      2.225657      0.000000      5.312346
 H      3.031184      0.893635      3.990942
 H      3.031184     -0.893635      3.990942
 H      0.128300      0.000000      5.276700
 H     -2.098735      0.000000      4.117340
 H     -2.164430      0.000000      1.624713
 H     -0.943212      0.000000     -0.541199
 H      3.383774      0.000000     -0.499280
 H      3.406891      0.000000      1.946806
```

Optimized geometry of ground state MQ in water, at the M06-2x/6-31G(d) level of theory

```
   21
scf done:  -516.223644
 C    -1.208723     0.000000     2.152383
 C    -0.003249     0.000000     1.396863
 C     1.226963     0.000000     2.133994
 N     1.191067     0.000000     3.507739
 C     0.045084     0.000000     4.190349
 C    -1.184428     0.000000     3.523696
 C     2.450809     0.000000     1.415339
 C     2.434835     0.000000     0.050728
 C     1.214860     0.000000    -0.758018
 C     0.009635     0.000000     0.001137
 O     1.270352     0.000000    -2.012681
 C     2.458598     0.000000     4.257444
 H     2.233338     0.000000     5.320825
 H     3.028800     0.893483     4.002562
 H     3.028800    -0.893483     4.002562
 H     0.121253     0.000000     5.269379
 H    -2.094394     0.000000     4.110378
 H    -2.155720     0.000000     1.620484
 H    -0.937568     0.000000    -0.531115
 H     3.374806     0.000000    -0.495288
 H     3.399894     0.000000     1.938504
```

Optimized geometry of ground state MQ in water, at the M06-2x/Sadlej level of theory

```
   21
scf done:  -516.353051
 C    -1.209137     0.000000     2.151416
 C    -0.002483     0.000000     1.401175
 C     1.224747     0.000000     2.134560
 N     1.191237     0.000000     3.507360
 C     0.048340     0.000000     4.186985
 C    -1.184388     0.000000     3.523759
 C     2.448354     0.000000     1.418366
 C     2.435200     0.000000     0.050979
 C     1.215338     0.000000    -0.748565
 C     0.010422     0.000000    -0.000576
 O     1.271386     0.000000    -2.014369
 C     2.456123     0.000000     4.253485
 H     2.230279     0.000000     5.319918
 H     3.031907     0.895353     3.997752
 H     3.031907    -0.895353     3.997752
 H     0.122201     0.000000     5.270411
 H    -2.096503     0.000000     4.114892
 H    -2.159253     0.000000     1.616995
 H    -0.941291     0.000000    -0.532885
 H     3.380075     0.000000    -0.495417
 H     3.400547     0.000000     1.943271
```

Optimized geometry of excited state MQ in gasphase, at the M06-2x/6-31G(d) level of theory

```
   21
scf done:  -516.123396
 C    -1.233802     0.000000     2.135936
 C    -0.016623     0.000000     1.442651
 C     1.214324     0.000000     2.113622
 N     1.206969     0.000000     3.521965
 C     0.006872     0.000000     4.199131
 C    -1.190181     0.000000     3.555159
 C     2.440814     0.000000     1.382638
 C     2.482724     0.000000     0.019575
 C     1.246287     0.000000    -0.751606
 C     0.021222     0.000000     0.009929
 O     1.219579     0.000000    -1.994523
 C     2.457123     0.000000     4.249574
 H     2.243296     0.000000     5.318475
 H     3.049932     0.890368     4.010066
 H     3.049932    -0.890368     4.010066
 H     0.087885     0.000000     5.279661
 H    -2.098918     0.000000     4.144806
 H    -2.171854     0.000000     1.595646
 H    -0.906831     0.000000    -0.553256
 H     3.419633     0.000000    -0.524221
 H     3.376627     0.000000     1.931972
```

Optimized geometry of excited state MQ in gasphase, at the M06-2x/Sadlej level of theory

```
   21
scf done:  -516.248069
 C    -1.235976     0.000000     2.136934
 C    -0.017816     0.000000     1.438897
 C     1.213864     0.000000     2.115574
 N     1.206047     0.000000     3.517913
 C     0.011488     0.000000     4.197595
 C    -1.191338     0.000000     3.555276
 C     2.441201     0.000000     1.386332
 C     2.485700     0.000000     0.020934
 C     1.249050     0.000000    -0.749951
 C     0.018688     0.000000     0.010619
 O     1.222257     0.000000    -1.991324
 C     2.454324     0.000000     4.245089
 H     2.239755     0.000000     5.316640
 H     3.051693     0.892392     4.005174
 H     3.051693    -0.892392     4.005174
 H     0.088030     0.000000     5.282199
 H    -2.100417     0.000000     4.151806
 H    -2.177732     0.000000     1.594577
 H    -0.913258     0.000000    -0.554587
 H     3.426932     0.000000    -0.524248
 H     3.380825     0.000000     1.936646
```

Optimized geometry of excited state MQ in water, at the M06-2x/6-31G(d) level of theory

```
   21
scf done:   -516.133970
 C     -1.243008      0.000000      2.138925
 C     -0.023379      0.000000      1.431671
 C      1.217229      0.000000      2.116495
 N      1.207455      0.000000      3.508209
 C      0.006729      0.000000      4.196986
 C     -1.193883      0.000000      3.552614
 C      2.443890      0.000000      1.389078
 C      2.485998      0.000000      0.019650
 C      1.253790      0.000000     -0.742908
 C      0.018477      0.000000      0.019535
 O      1.222518      0.000000     -1.989792
 C      2.461231      0.000000      4.244103
 H      2.241786      0.000000      5.310405
 H      3.049321      0.891058      4.003414
 H      3.049321     -0.891058      4.003414
 H      0.094588      0.000000      5.275715
 H     -2.100964      0.000000      4.144959
 H     -2.183016      0.000000      1.601165
 H     -0.908129      0.000000     -0.546932
 H      3.426705      0.000000     -0.518502
 H      3.378351      0.000000      1.939061
```

Optimized geometry of excited state MQ in water, at the M06-2x/Sadlej level of theory

```
   21
scf done:   -516.260074
 C     -1.245456      0.000000      2.140185
 C     -0.025772      0.000000      1.427419
 C      1.217528      0.000000      2.119355
 N      1.206570      0.000000      3.502578
 C      0.010765      0.000000      4.194874
 C     -1.195191      0.000000      3.553296
 C      2.445251      0.000000      1.394023
 C      2.488990      0.000000      0.021198
 C      1.257423      0.000000     -0.739613
 C      0.015551      0.000000      0.020982
 O      1.226365      0.000000     -1.986815
 C      2.458330      0.000000      4.238400
 H      2.237335      0.000000      5.307232
 H      3.050543      0.893244      3.996903
 H      3.050543     -0.893244      3.996903
 H      0.096826      0.000000      5.277764
 H     -2.103158      0.000000      4.151919
 H     -2.189841      0.000000      1.601250
 H     -0.914598      0.000000     -0.547970
 H      3.434024      0.000000     -0.518555
 H      3.382982      0.000000      1.945939
```

2 Definition of bond charges and the resulting dipole moment

The net charges on atomic sites i of a neutral molecule can be distributed among the bonds connected to the site so that

$$q_i = \sum_{j=1}^{N_b} q_{b(ij)} \tag{2}$$

where $q_{b(ij)}$ are the directed contributions to the charge q_i. Since

$$q_{b(ij)} = -q_{b(ji)} \tag{3}$$

we choose to use the non-unique set of bond charges where $i < j$ for all further calculation. Any other set can be chosen. For a minimal working example, Fig S1, we get

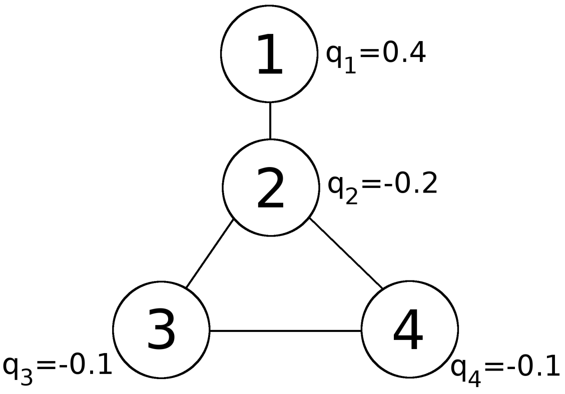

Figure S1: Structure and charges of an artificial molecule

$$\begin{aligned}
q_{b(12)} \quad & = q_1 \\
-q_{b(12)} + q_{b(23)} \quad + q_{b(24)} & = q_2 \\
- q_{b(23)} + q_{b(34)} \quad & = q_3 \\
-q_{b(34)} + q_{b(24)} & = q_4 \\
q_{b(23)} + q_{b(34)} - q_{b(24)} & = 0
\end{aligned} \tag{4}$$

where the first four rows arise from Eqn (2) and (3), and the last from the constraint that bond charges within a ring add up to 0. Reformulating Eqn (4) and using the respective q_i from Fig. S1 yields

$$\begin{pmatrix} 1 & 0 & 0 & 0 \\ -1 & 1 & 0 & 1 \\ 0 & -1 & 1 & 0 \\ 0 & 0 & -1 & -1 \\ 0 & 1 & 1 & -1 \end{pmatrix} \cdot \begin{pmatrix} q_{b(12)} \\ q_{b(23)} \\ q_{b(34)} \\ q_{b(24)} \end{pmatrix} = \begin{pmatrix} 0.4 \\ -0.2 \\ -0.1 \\ -0.1 \\ 0 \end{pmatrix} \tag{5}$$

This set of equations can be solved, yielding for our minimal working example

$$\begin{pmatrix} q_{b(12)} \\ q_{b(23)} \\ q_{b(34)} \\ q_{b(24)} \end{pmatrix} = \begin{pmatrix} 0.4 \\ 0.1 \\ 0.0 \\ 0.1 \end{pmatrix} \tag{6}$$

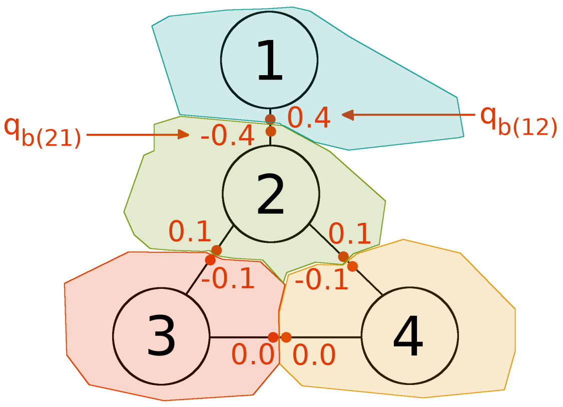

Figure S2: Bond charges of an artificial molecule

as also depicted in Fig. S2. The dipole (charge transfer contribution) with respect to the atomic site is then

$$\mu_{ic} = \sum_{j=1}^{N_b} q_{b(ij)}(R_i - R_{b(ij)}) \tag{7}$$

where R_i is the nuclear position, and $R_{b(ij)}$ the bond critical point, or here simply $\frac{R_i+R_j}{2}$. Let us evalute μ_{ic} for the 2-dimensional coordinates

$$\begin{pmatrix} R_1 \\ R_2 \\ R_3 \\ R_4 \end{pmatrix} = \begin{pmatrix} 0 & 1 \\ 0 & 0 \\ -1 & -1 \\ 1 & -1 \end{pmatrix} \tag{8}$$

$$\mu_{1c} = q_{b(12)}(R_1 - R_{b(12)}) = 0.4\left[\begin{pmatrix} 0 \\ 1 \end{pmatrix} - \begin{pmatrix} 0 \\ 0.5 \end{pmatrix}\right] = \begin{pmatrix} 0 \\ 0.2 \end{pmatrix} \tag{9}$$

$$\begin{gathered}\mu_{2c} = -q_{b(12)}(R_2 - R_{b(12)}) + q_{b(23)}(R_2 - R_{b(23)}) + q_{b(24)}(R_2 - R_{b(24)}) = \\ -0.4\left[\begin{pmatrix} 0 \\ 0 \end{pmatrix} - \begin{pmatrix} 0 \\ 0.5 \end{pmatrix}\right] + 0.1\left[\begin{pmatrix} 0 \\ 0 \end{pmatrix} - \begin{pmatrix} -0.5 \\ -0.5 \end{pmatrix}\right] + 0.1\left[\begin{pmatrix} 0 \\ 0 \end{pmatrix} - \begin{pmatrix} 0.5 \\ -0.5 \end{pmatrix}\right] = \begin{pmatrix} 0 \\ 0.3 \end{pmatrix}\end{gathered} \tag{10}$$

$$\mu_{3c} = \begin{pmatrix} 0.05 \\ 0.05 \end{pmatrix} \tag{11}$$

$$\mu_{4c} = \begin{pmatrix} -0.05 \\ 0.05 \end{pmatrix} \tag{12}$$

Summing up all contributions leads to $\mu_c = \sum_i \mu_{ic} = \begin{pmatrix} 0 \\ 0.6 \end{pmatrix}$. This result can also be obtained via the traditional definition of the dipole moment $\mu_c = \sum_i q_i R_i = \begin{pmatrix} 0 \\ 0.6 \end{pmatrix}$. Calculating μ_{ic} at different external fields and numerically differentiating finally yields α_{ic}.

3 Romberg differentiation procedure

The Romberg differentiation procedure is based on the recursive formula

$$P^{p,k} = \frac{4^p P^{p-1,k} - P^{p-1,k+1}}{4^p - 1} \tag{13}$$

with k=1,2 ..., p the number of Romberg iteration and P the current differential. Before the first iteration (for p=0), the differential

$$P^{0,k} = \frac{d^2 f}{dx} = \frac{f(-2^k h) + f(2^k h) - 2f(0)}{(2^k h)^2} \tag{14}$$

is evaluated directly for multiple values of k.
We applied an electric field of magnitude 0.0008, 0.0016, 0.0032, 0.0064 and 0.0128 au in each of the positive and negative x, y and z directions and obtained the resultant energy (ie 33 separate calculations) For example, in G09 to apply an electric field in the x direction of 0.0008 au include the keywords density=current and field=x+8. Then for a second derivative with k=1 and h=0.0004 au

$$\alpha_{xx} = \frac{\partial E^2}{\partial x \partial x} = \frac{E(-2h) + E(2h) - Ef(0)}{(2h)^2} \tag{15}$$

$$\alpha_{xx} = \frac{E(-0.0008) + E(0.0008) - 2E(0.0000)}{(0.0008)^2} \tag{16}$$

This result is iteratively improved by usage of the recursive formula, Eqn (13).

4 Python script

A python script was developed to read the output of GDMA (multipole rank 1) and transform the atomic dipoles and charges to atomic and molecular polarizabilities. The script is reported at the end of this section and can also be downloaded from http://www.mdy.univie.ac.at/python-stuff/atomic_polarizabilities.py. It requires *python3*, as well as the packages *numpy*, *re* and *sys*. To use the script, six output files are needed, where an electric field of the same strength was applied in the positive and negative x, y and z direction respectively. Furthermore, the script requires an input file, where the connectivity of the atoms, as well as optional rings can be specified. For the minimal working example from **Section 2**, the input file reads as:

```
bond 1 2
bond 2 3
bond 3 4
bond 4 2
ring 2 3 4
```

There is no order of indices that needs to be taken care of ('bond 1 2' is equivalent to 'bond 2 1'). The atom numbering must be the same as in GDMA (which is the same atom numbering as in Gaussian09). Then, execute

```
$ python3 atomic_polarizabilities.py
```

The program asks where the six output files and the connectivity input files are located. Per default, 'x.out' is the GDMA output for field in positive x direction, 'mx.out' for a field in negative x direction, and so on, all located in the subdirectory 'out'. The connectivity file is per default named as 'connec.inp'. Other directories and names can be specified. The program now parses through the files, and lists the coordinates, charges and dipole moments of each atom (atom names and order as in GDMA output). Then, the field strength needs to be specified. For example, when ± 0.0008 au were applied (Gaussian option 'field=x+8'), enter 0.0008. Furthermore the script asks whether atomic polarizabilities should be given in [au] or [Å^3]. The atomic polarizabilities from charge transfer and polarization are then printed.

In the following, the Python script to calculate atomic polarizabilities from atomic charges and dipole moments is given.

```
import numpy as np
import sys
import re


print("---------------------------------------------------------")
print("---------------------Welcome-----------------------------")
print("---------------------------------------------------------")

#===============================================================================================

#This part reads the input files for the connectivity
#===============================================================================================

print("Read connectivity from default directory?")
print("./connec.inp")
answer=input("........... yes/no   ")     # Ask whether to use default directory / enter
    own path
if answer=="yes":
    print("Reading file...")
    try:
        read_input = open("connec.inp",'r')
    except FileNotFoundError:
        print("---------------------------------------------------------")
        sys.exit("File not found. Please check the corresponding paths.")
else:
    if answer=="no":
        filename=input("Enter file name...")
        print("Reading file...")
        try:
            read_input = open(filename,'r')
        except FileNotFoundError:
            print("---------------------------------------------------------")
            sys.exit("File not found. Please check the corresponding paths.")
    else:
        print("---------------------------------------------------------")
        sys.exit("Unknown option")

tmp=''.join([n for n in read_input.readlines()][:])          # Read input file into
    variable
read_input.close()
bond=re.findall(r"bond\s*\d+\s+\d+\s",tmp)                   # Search for all bonds
bonds=len(bond)
ring=re.findall(r"ring[\s\d]+",tmp)                          # Search for all rings
rings=len(ring)

if bonds==0:
    sys.exit("No bonds found, check input format.")
if len(re.findall(r"[\S ]+\n",tmp))>bonds+rings:
    answer=input("Not all lines in the input file contained readable information. Continue
         nevertheless?  yes/no   ")
    if answer=="yes":
        print("---------------------------------------------------------")
```

```
        print("Continue... if program fails at a later point, check connectivity input
            file.")
    else:
        if answer=="no":
            print("---------------------------------------------------------")
            sys.exit("Program stopped. Check connectivity input file.")
        else:
            print("---------------------------------------------------------")
            sys.exit("Unknown option")
print("Molecule contains "+str(bonds)+" bonds and "+str(rings)+" rings.")

#=============================================================================================

#This part reads the GDMA output for six files (field in positive and
#negative x, y and z direction) and creates arrays for atoms names ("name"),
#coordinates ("c"), charges ("q") and dipoles ("d")
#=============================================================================================


print("Read GDMA output from default directory?")
print("./out/x.out")
print("./out/y.out")
print("./out/z.out")
print("./out/mx.out")
print("./out/my.out")
print("./out/mz.out")
answer=input("........... yes/no   ")       # Ask whether to use default directory /
    enter own path
if answer=="yes":
    print("Reading files...")
    try:
        read_input_x = open("out/x.out",'r')
        read_input_y = open("out/y.out",'r')
        read_input_z = open("out/z.out",'r')
        read_input_mx = open("out/mx.out",'r')
        read_input_my = open("out/my.out",'r')
        read_input_mz = open("out/mz.out",'r')
    except FileNotFoundError:
        print("---------------------------------------------------------")
        sys.exit("File not found. Please check the corresponding paths.")
else:
    if answer=="no":
        print("Enter file names for field in...")
        x=input("...positive x direction:   ")
        y=input("...positive y direction:   ")
        z=input("...positive z direction:   ")
        mx=input("...negative x direction:   ")
        my=input("...negative y direction:   ")
        mz=input("...negative z direction:   ")
        print("Reading files...")
        try:
            read_input_x = open(x,'r')
            read_input_y = open(y,'r')
            read_input_z = open(z,'r')
            read_input_mx = open(mx,'r')
            read_input_my = open(my,'r')
            read_input_mz = open(mz,'r')
        except FileNotFoundError:
            print("---------------------------------------------------------")
            sys.exit("File not found. Please check the corresponding paths.")
    else:
        print("---------------------------------------------------------")
        sys.exit("Unknown option")


tmp=[''.join([n for n in read_input_x.readlines()][:]),  # Read input files into variable
     ''.join([n for n in read_input_y.readlines()][:]),
     ''.join([n for n in read_input_z.readlines()][:]),
     ''.join([n for n in read_input_mx.readlines()][:]),
     ''.join([n for n in read_input_my.readlines()][:]),
     ''.join([n for n in read_input_mz.readlines()][:])]
```

```
read_input_x.close()
read_input_y.close()
read_input_z.close()
read_input_mx.close()
read_input_my.close()
read_input_mz.close()

coordinates=re.findall(r"[A-Z]\S* *x =\s*\S*\s*y =\s*\S*\s*z =\s*\S*", tmp[0], flags=0)
atoms=len(coordinates)                        # Number of atoms
q=np.zeros((atoms+rings,6))                   # Create empty charge array (+zero rows for
     ring conditions)
c=np.zeros((atoms,3))                         # Create empty coordinates array
d=np.zeros((atoms,3,6))                       # Create empty atomic dipole array
name=[0]*atoms                                # Create empty array for atom names
for i in range(atoms):
    name[i]=coordinates[i][:int(re.search(r"\S*",coordinates[i]).end())]    # Read atom
        names from file
    c[i,0]=float(re.findall(r"[ -]\d*[.]\d*",coordinates[i])[0])            # Read x
        coordinates from file
    c[i,1]=float(re.findall(r"[ -]\d*[.]\d*",coordinates[i])[1])            # Read y
        coordinates from file
    c[i,2]=float(re.findall(r"[ -]\d*[.]\d*",coordinates[i])[2])            # Read z
        coordinatas from file
for j in range(6):
    charges=re.findall(r"Q00\s*\S*\s*\S*", tmp[j], flags=0)[:atoms]
    dipoles=re.findall(r"\|Q1\| =.*", tmp[j], flags=0)[:atoms]
    for i in range(atoms):
        q[i,j]=float(re.findall(r"[ -]\d*[.]\d*",charges[i])[0])          # Read charges
        if re.search(r"Q11c =\s*[ -]\d*[.]\d*",dipoles[i]):        # Look for x, y and z
            output (if zero, there is not respective output in GMDA!)
            d[i,0,j]=float(re.findall(r"[ -]\d*[.]\d*",re.findall(r"Q11c =\s*[ -]\d*[.]\d
                *",dipoles[i])[0])[0])          # atomic dipole x
        if re.search(r"Q11s =\s*[ -]\d*[.]\d*",dipoles[i]):
            d[i,1,j]=float(re.findall(r"[ -]\d*[.]\d*",re.findall(r"Q11s =\s*[ -]\d*[.]\d
                *",dipoles[i])[0])[0])          # atomic dipole y
        if re.search(r"Q10  =\s*[ -]\d*[.]\d*",dipoles[i]):
            d[i,2,j]=float(re.findall(r"[ -]\d*[.]\d*",re.findall(r"Q10  =\s*[ -]\d*[.]\d
                *",dipoles[i])[0])[0])          # atomic dipole z

print("%4s %7s %7s %7s %7s %7s %7s  %7s %7s %7s " % ("Name","x","y","z","q(Fx)","q(Fy)","q
    (Fz)","q(-Fx)","q(-Fy)","q(-Fz)"))
for i in range(atoms):
    print("%4s %7.4f %7.4f %7.4f %7.4f %7.4f %7.4f %7.4f %7.4f %7.4f " % (name[i],c[i,0],c
        [i,1],c[i,2],q[i,0],q[i,1],q[i,2],q[i,3],q[i,4],q[i,5]))
print("")
print("%4s %7s %7s %7s %7s %7s %7s  %7s %7s %7s " % ("Name","dx(Fx)","dy(Fx)","dz(Fx)","dx
    (Fy)","dy(Fy)","dz(Fy)","dx(Fz)","dy(Fz)","dz(Fz)"))
for i in range(atoms):
    print("%4s %7.4f %7.4f %7.4f %7.4f %7.4f %7.4f %7.4f %7.4f %7.4f " % (name[i],d[i
        ,0,0],d[i,1,0],d[i,2,0],d[i,0,1],d[i,1,1],d[i,2,1],d[i,0,2],d[i,1,2],d[i,2,2]))
print("")
print("%4s %7s %7s %7s %7s %7s %7s  %7s %7s %7s " % ("Name","dx(-Fx)","dy(-Fx)","dz(-Fx)
    ","dx(-Fy)","dy(-Fy)","dz(-Fy)","dx(-Fz)","dy(-Fz)","dz(-Fz)"))
for i in range(atoms):
    print("%4s %7.4f %7.4f %7.4f %7.4f %7.4f %7.4f %7.4f %7.4f %7.4f " % (name[i],d[i
        ,0,3],d[i,1,3],d[i,2,3],d[i,0,4],d[i,1,4],d[i,2,4],d[i,0,5],d[i,1,5],d[i,2,5]))
print("")


#==============================================================================================

#   CHARGE TRANSFER
#==============================================================================================

#This part calculates the connectivity matrix "n", the matrix with indices
#of each bond charge "index" the bond charges "b" and then the atomic
#dipoles "mu" and the polarizability "a" for the charge transfer
#==============================================================================================

print("")
```

```
print("---------------------------------------------------------")
print("------------------Starting calculation-------------------")
print("---------------------------------------------------------")


n=np.zeros((atoms,atoms))                                    # Create empty
    connectivity matrix
for i in range(bonds):
    first=int(re.findall(r"\d+",bond[i])[0])-1               # Read atom numbers of
        each bond
    second=int(re.findall(r"\d+",bond[i])[1])-1
    if first<second:                                         # Write entry in
        antisymmetric connectivity matrix
        n[first,second]=1
        n[second,first]=-1
    else:
        n[first,second]=-1
        n[second,first]=1

index=np.zeros((bonds,2))                                    # Create index matrix (
    which bond charge contains which atoms)
ctr=0
for i in range(atoms):
    for j in range(i,atoms):
        if n[i,j]==1:
            index[ctr,0]=i
            index[ctr,1]=j
            ctr+=1

a=np.zeros((atoms+rings,bonds))                              # Create matrix for
    linear equations ab=q
for i in range(atoms):
    for j in range(bonds):
        if i==index[j,0]:
            a[i,j]=1
        if i==index[j,1]:
            a[i,j]=-1

for i in range(rings):                                       # Write ring conditions
    into matrix a
    structure=re.findall(r"\d+",ring[i])
    for j in range(len(structure)):
        if j ==len(structure)-1:
            k=0
        else:
            k=j+1
        first=int(structure[j])-1
        second=int(structure[k])-1
        factor=1
        if second<first:
            save=first
            first=second
            second=save
            factor=-1
        # Find the correct bond charge which is involved in the ring
        element=np.where(np.all(index==np.array([[first,second]]),axis=1))[0][0]
        a[atoms+i,element]=factor                             # Write entry in a

#Calculate bond charges and mu
mu=np.zeros((atoms,3,6))
for k in range(6):
    b=np.linalg.lstsq(a,q[:,k])[0]                            # Solve linear
        equations ab=q for b


    for i in range(atoms):                                    # Calculate atomic
        dipole from charge transfer as sum of bond charges times vector of the
        for j in range(bonds):                                # atom in direction of
            each bond
            if i==index[j,0]:
                mu[i,:,k]+=(c[i]-(c[i]+c[int(index[j,1])])/2)*b[j]
```

```
            if i==index[j,1]:
                mu[i,:,k]+=(c[i]-(c[i]+c[int(index[j,0])])/2)*b[j]*(-1)

#Calculate polarizability
field=float(input("Enter field strength (in au):   "))
answer=input("Do you want to change the default units (au.) of the output to angstrom^3?
    yes/no   ")
if answer=="yes":
    unit=0.529177249**3                                            #transform au^3 into A
        ^3
else:
    if answer=="no":
        unit=1
    else:
        print("----------------------------------------------------------")
        sys.exit("unknown option")

a_xx=(mu[:,0,0]-mu[:,0,3])/(2*field)*1.889725989*(-1)*unit   #numeric differentiation,
    yields polarizability
a_yy=(mu[:,1,1]-mu[:,1,4])/(2*field)*1.889725989*(-1)*unit
a_zz=(mu[:,2,2]-mu[:,2,5])/(2*field)*1.889725989*(-1)*unit
a_tot_c=(a_xx+a_yy+a_zz)/3
print("")
print("Results:")
print("")
print("Charge transfer contribution:")
print("%4s %7s %7s %7s %7s" % ("Name","a_xx","a_yy","a_zz","a_tot"))
for i in range(atoms):
    print("%4s %7.2f %7.2f %7.2f %7.2f " % (name[i],a_xx[i],a_yy[i],a_zz[i],a_tot_c[i]))
print("Summed up contributions:")
print(a_tot_c[:].sum())

#=========================================================================================
#   Atomic polarizability
#=========================================================================================
#This part calculates the polarizability arising from non-uniform
# distribution of electrons around the core
#=========================================================================================

a_xx=(d[:,0,0]-d[:,0,3])/(2*field)*(-1)*unit     #numeric differentiation, yields
    polarizability
a_yy=(d[:,1,1]-d[:,1,4])/(2*field)*(-1)*unit
a_zz=(d[:,2,2]-d[:,2,5])/(2*field)*(-1)*unit
a_tot_p=(a_xx+a_yy+a_zz)/3
print("Polarization contribution:")
print("%4s %7s %7s %7s %7s" % ("Name","a_xx","a_yy","a_zz","a_tot"))
for i in range(atoms):
    print("%4s %7.2f %7.2f %7.2f %7.2f " % (name[i],a_xx[i],a_yy[i],a_zz[i],a_tot_p[i]))
print("Summed up contributions:")
print(a_tot_p[:].sum())

print("Total polarizability:")
print("%4s %7s" % ("Name","a"))
for i in range(atoms):
     print("%4s %7.2f " % (name[i],a_tot_p[i]+a_tot_c[i]))
print("Summed up contributions:")
print(a_tot_p[:].sum()+a_tot_c[:].sum())

print("------------------------------------------------------")
print("-------------------------Goodbye-------------------------")
print("------------------------------------------------------")
```

5 Influence of the cavity radius in continuum models on the polarizability

In implicit solvent models the solute is placed into a cavity, surrounded by an continuous dielectric medium mimicking the solvent. Depending on the cavity radius, the charges on the cavity surface move further away from the solute, thus weakening the interaction between solute and solvent. A infinitely large cavity corresponds thus to a vacuum calculation. The dipole moments and polarizabilities of the solute vary largely with the scaling factor determining the difference between van der Waals radii and the actual cavity volume, Table S2.

Table S2: Dipole moments in [D] and polarizabilities in [Å^3] of MQ for different scaling factors f for the cavity radius

f	1.1	1.2	2.0	∞
$\mu_{\mathbb{S}_0}$	16.9	16.0	12.7	10.8
$\mu_{\mathbb{S}_1}$	9.7	9.3	7.9	6.9
$\alpha_{\mathbb{S}_0}$	30.8	29.8	24.9	21.9
$\alpha_{\mathbb{S}_1}$	31.7	29.9	23.7	20.4

The default scaling factor, 1.1, was used for all calculations with PCM solvents, as well as 1.0 for SMD solvents.

6 Atomic polarizabilities of coumarin 153

The atomic polarizability of C153 from charge transfer and polarization is reported in Table S3.

Table S3: Atomic polarizability $\alpha_{p,i}$ and $\alpha_{c,i}$ in [Å^3] of C153 in PCM water. Atom labeling as in the main article.

	Ground state			Excited state			$\Delta\alpha$		Ground state			Excited state			$\Delta\alpha$
i	$\alpha_{p,i}$	$\alpha_{c,i}$	α_i	$\alpha_{p,i}$	$\alpha_{c,i}$	α_i	$\Delta\alpha_i$	i	$\alpha_{p,i}$	$\alpha_{c,i}$	α_i	$\alpha_{p,i}$	$\alpha_{c,i}$	α_i	$\Delta\alpha_i$
C_1	0.30	1.39	1.69	0.30	1.50	1.79	6%	O_{15}	1.08	0.59	1.67	1.24	0.64	1.90	13%
C_2	0.49	1.93	2.42	0.41	2.09	2.50	4%	F_{17A}	0.49	0.21	0.70	0.52	0.21	0.73	5%
C_3	0.30	1.81	2.10	0.34	1.91	2.25	7%	F_{17B}	0.49	0.19	0.70	0.52	0.21	0.73	5%
N_4	0.47	1.47	1.94	0.39	1.62	2.00	3%	F_{17C}	0.53	0.24	0.76	0.55	0.25	0.82	7%
C_5	0.27	1.22	1.48	0.28	1.27	1.56	5%	H_7	0.18	0.15	0.33	0.18	0.15	0.33	-1%
C_6	0.33	1.24	1.57	0.33	1.36	1.69	7%	H_{16}	0.24	0.21	0.44	0.27	0.19	0.47	7%
C_7	0.46	1.78	2.25	0.56	1.90	2.46	10%	H_{13A}	0.24	0.13	0.37	0.22	0.13	0.36	-7%
C_8	0.50	2.16	2.67	0.27	2.34	2.61	-3%	H_{13B}	0.27	0.16	0.43	0.28	0.16	0.43	1%
C_9	0.28	2.22	2.50	0.40	2.55	2.95	18%	H_{12A}	0.30	0.18	0.47	0.30	0.19	0.49	1%
C_{10}	0.47	2.18	2.65	0.37	2.45	2.82	6%	H_{12B}	0.27	0.15	0.41	0.25	0.15	0.40	-2%
C_{11}	0.27	1.23	1.50	0.28	1.26	1.54	3%	H_{11A}	0.30	0.16	0.46	0.31	0.16	0.47	5%
C_{12}	0.31	1.36	1.67	0.31	1.36	1.69	0%	H_{11B}	0.28	0.18	0.47	0.27	0.19	0.46	-2%
C_{13}	0.28	1.32	1.59	0.28	1.39	1.67	6%	H_{5A}	0.28	0.18	0.47	0.27	0.19	0.46	-2%
O_{14}	0.53	1.44	1.97	0.59	1.48	2.09	6%	H_{5B}	0.31	0.16	0.49	0.31	0.18	0.49	1%
C_{15}	0.22	1.76	1.99	0.33	1.93	2.25	13%	H_{6A}	0.31	0.19	0.50	0.30	0.19	0.49	-3%
C_{16}	0.65	1.39	2.03	0.71	1.36	2.07	2%	H_{6B}	0.27	0.15	0.41	0.25	0.13	0.40	-3%
C_{17}	0.25	2.12	2.37	0.49	2.19	2.68	13%	H_{1A}	0.28	0.16	0.46	0.28	0.16	0.44	-2%
C_{18}	0.01	1.13	1.14	0.00	1.26	1.26	11%	H_{1B}	0.28	0.16	0.43	0.27	0.16	0.43	-2%
total									12.7	32.8	45.5	13.2	34.8	48.2	6%

7 Dipole moments and polarizabilities of MQ and C153 in atomic units

Table S4: Dipole moments μ in [au] and polarizabilities α, as well as polarizability anisotropy γ in [au] of MQ in gas phase and water using different functionals, basis sets and literature values.

	M06-2X		ωB97xD		B3LYP-D3BJ	
	Sadlej		aug-cc-pVTZ		6-311G(d,p)	
	vacuum	water	vacuum	water	vacuum	water
$\mu_{\mathbb{S}_0}$	4.26	6.65	4.32	6.71	3.99	6.03
$\mu_{\mathbb{S}_1}$	2.70	3.80	2.68	3.80	2.82	3.65
$\alpha_{\mathbb{S}_0}$	148	208	148	209	134	187
$\alpha_{\mathbb{S}_1}$	138	214	137	216	118	165
$\gamma_{\mathbb{S}_0}$	119	170	119	169	128	190
$\gamma_{\mathbb{S}_1}$	91	165	91	171	96	143

Table S5: Dipole moments in [au] and polarizabilities in [au] for MQ and C153 in different solvents using the PCM and SMD implicit solvent models. For the explicit water models (termed 'expl.'), 'opt.' refers to a calculation at a single, optimized geometry, whereas 'rep.' refers to the average over ten snapshots from MD simulations without optimization (95% confidence interval given).

		PCM				SMD		expl.+PCM	
	Vacuum	Water	MeOH	EtOH	C_2mimBF_4	Water	C_2mimBF_4	opt.	rep.
ϵ	1	78.4	32.6	24.9	12.9	78.4	12.9		
MQ:									
$\mu_{\mathbb{S}_0}$	4.26	6.65	6.57	6.53	6.37	7.57	6.88	5.7	5.6±0.5
$\mu_{\mathbb{S}_1}$	2.70	3.80	3.76	3.74	3.66	4.36	3.96	3.3	3.3±0.4
$\alpha_{\mathbb{S}_0}$	148	208	206	205	201	222	214	201	212±4
$\alpha_{\mathbb{S}_1}$	138	214	211	209	203	245	225	219	232±5
$\Delta\alpha$	-7%	+3%	+2%	+2%	+1%	+10%	+5%	+9%	+9%
C153:									
$\mu_{\mathbb{S}_0}$	2.75	3.95	3.91	3.89			4.03		
$\mu_{\mathbb{S}_1}$	4.86	7.42	7.34	7.29			7.63		
$\alpha_{\mathbb{S}_0}$	213	307	303	301			315		
$\alpha_{\mathbb{S}_1}$	235	325	321	319			331		
$\Delta\alpha$	+10%	+6%	+6%	+6%			+5%		

Table S6: Atomic polarizability α_i and the contributions from polarization, $\alpha_{p,i}$ and charge transfer, $\alpha_{c,i}$ of MQ, MQ·3H_2O and C153 in PCM water in the ground and excited state. $\Delta\alpha_i$ describes the change of atomic polarizability upon excitation. Atom labeling as in the main article.

	1MQ in PCM water							1MQ·3H_2O in PCM water						
	Ground state			Excited state			$\Delta\alpha$	Ground state			Excited state			$\Delta\alpha$
i	$\alpha_{p,i}$	$\alpha_{c,i}$	α_i	$\alpha_{p,i}$	$\alpha_{c,i}$	α_i	$\Delta\alpha_i$	$\alpha_{p,i}$	$\alpha_{c,i}$	α_i	$\alpha_{p,i}$	$\alpha_{c,i}$	α_i	$\Delta\alpha_i$
C_N	2.2	5.8	8.0	2.2	6.3	8.5	6%	2.2	5.8	8.0	2.2	6.3	8.5	7%
H_{N1}	1.6	1.0	2.7	1.8	1.1	2.9	9%	1.6	1.0	2.6	1.8	1.1	2.9	10%
H_{N2}	1.6	0.9	2.5	1.9	1.0	2.9	17%	1.5	0.9	2.4	1.9	1.0	2.8	16%
H_{N3}	1.6	0.9	2.5	1.9	1.0	2.9	17%	1.5	0.9	2.4	1.9	1.0	2.8	17%
N_1	2.0	13.5	15.5	2.6	13.6	16.2	5%	2.0	12.6	14.6	2.6	13.8	16.4	12%
C_2	4.0	9.2	13.2	5.0	9.6	14.6	11%	3.6	8.7	12.3	4.8	9.7	14.5	18%
H_2	1.8	1.5	3.3	2.2	1.6	3.8	15%	1.7	1.4	3.1	2.2	1.6	3.7	21%
C_3	4.3	9.0	13.2	4.9	9.8	14.7	11%	4.3	8.7	13.0	9.8	4.8	14.6	12%
H_3	2.0	1.5	3.4	2.2	1.5	3.7	8%	1.9	1.4	3.3	2.1	1.5	3.7	10%
C_4	3.7	11.4	15.1	4.7	11.9	16.6	10%	3.5	10.7	14.1	4.7	12.0	16.7	18%
H_4	1.8	1.2	3.0	2.2	1.4	3.5	17%	1.7	1.2	2.9	2.1	1.3	3.4	18%
C_{4A}	2.6	15.5	18.2	3.3	16.8	20.1	10%	2.7	14.3	17.0	3.2	17.0	20.2	19%
C_5	4.9	13.1	18.1	3.7	12.8	16.5	-8%	4.5	11.9	16.4	3.6	12.8	16.4	0%
H_5	2.1	1.3	3.4	1.8	1.2	3.0	-13%	1.4	1.0	2.4	1.1	0.9	2.1	-15%
C_6	2.4	14.3	16.6	2.3	13.0	15.3	-8%	2.2	13.8	16.0	2.1	13.9	15.9	0%
O_6	10.0	4.9	14.9	8.1	4.6	12.7	-15%	5.9	9.5	15.4	5.3	8.2	13.5	-12%
C_7	4.4	10.4	14.8	5.1	10.6	15.7	7%	4.0	9.4	13.4	5.1	10.8	14.8	18%
H_7	2.2	1.5	3.7	2.3	1.6	3.9	5%	1.3	1.1	2.4	2.0	1.5	3.5	46%
C_8	4.0	11.7	15.6	3.9	12.2	16.1	3%	3.9	10.8	14.7	3.9	12.4	16.3	11%
H_8	1.6	1.1	2.8	1.6	1.1	2.7	-2%	1.6	1.1	2.7	1.6	1.1	2.7	0%
C_{8A}	2.9	14.8	17.6	2.6	15.5	18.2	3%	2.7	13.7	16.4	2.6	15.7	18.3	12%
total	63	145	208	66	148	214	3%	57	140	196	62	153	215	10%

C153 in PCM water															
	Ground state			Excited state			$\Delta\alpha$		Ground state			Excited state			$\Delta\alpha$
i	$\alpha_{p,i}$	$\alpha_{c,i}$	α_i	$\alpha_{p,i}$	$\alpha_{c,i}$	α_i	$\Delta\alpha_i$	i	$\alpha_{p,i}$	$\alpha_{c,i}$	α_i	$\alpha_{p,i}$	$\alpha_{c,i}$	α_i	$\Delta\alpha_i$
C_1	2.0	9.4	11.4	2.0	10.1	12.1	6%	O_{15}	7.3	4.0	11.3	8.4	4.3	12.8	13%
C_2	3.3	13.0	16.3	2.8	14.1	16.9	4%	F_{17A}	3.3	1.4	4.7	3.5	1.4	4.9	5%
C_3	2.0	12.2	14.2	2.3	12.9	15.2	7%	F_{17B}	3.3	1.3	4.7	3.5	1.4	4.9	5%
N_4	3.2	9.9	13.1	2.6	10.9	13.5	3%	F_{17C}	3.6	1.6	5.1	3.7	1.7	5.5	7%
C_5	1.8	8.2	10.0	1.9	8.6	10.5	5%	H_7	1.2	1.0	2.2	1.2	1.0	2.2	-1%
C_6	2.2	8.4	10.6	2.2	9.2	11.4	7%	H_{16}	1.6	1.4	3.0	1.8	1.3	3.2	7%
C_7	3.1	12.0	15.2	3.8	12.8	16.6	10%	H_{13A}	1.6	0.9	2.5	1.5	0.9	2.4	-7%
C_8	3.4	14.6	18.0	1.8	15.8	17.6	-3%	H_{13B}	1.8	1.1	2.9	1.9	1.1	2.9	1%
C_9	1.9	15.0	16.9	2.7	17.2	19.9	18%	H_{12A}	2.0	1.2	3.2	2.0	1.3	3.3	1%
C_{10}	3.2	14.7	17.9	2.5	16.5	19.0	6%	H_{12B}	1.8	1.0	2.8	1.7	1.0	2.7	-2%
C_{11}	1.8	8.3	10.1	1.9	8.5	10.4	3%	H_{11A}	2.0	1.1	3.1	2.1	1.1	3.2	5%
C_{12}	2.1	9.2	11.3	2.1	9.2	11.4	0%	H_{11B}	1.9	1.2	3.2	1.8	1.3	3.1	-2%
C_{13}	1.9	8.9	10.7	1.9	9.4	11.3	6%	H_{5A}	1.9	1.2	3.2	1.8	1.3	3.1	-2%
O_{14}	3.6	9.7	13.3	4.0	10.0	14.1	6%	H_{5B}	2.1	1.1	3.3	2.1	1.2	3.3	1%
C_{15}	1.5	11.9	13.4	2.2	13.0	15.2	13%	H_{6A}	2.1	1.3	3.4	2.0	1.3	3.3	-3%
C_{16}	4.4	9.4	13.7	4.8	9.2	14.0	2%	H_{6B}	1.8	1.0	2.8	1.7	0.9	2.7	-3%
C_{17}	1.7	14.3	16.0	3.3	14.8	18.1	13%	H_{1A}	1.9	1.1	3.1	1.9	1.1	3.0	-2%
C_{18}	0.1	7.6	7.7	0.0	8.5	8.5	11%	H_{1B}	1.9	1.1	2.9	1.8	1.1	2.9	-2%
total									86	221	307	89	235	325	6%
